# *YWHAG* Deficiency Disrupts the EMT‐Associated Network to Induce Oxidative Cell Death and Prevent Metastasis

**DOI:** 10.1002/advs.202301714

**Published:** 2023-09-27

**Authors:** Jeannie Xue Ting Lee, Wei Ren Tan, Zun Siong Low, Jia Qi Lee, Damien Chua, Wisely Duan Chi Yeo, Benedict See, Marcus Ivan Gerard Vos, Tomohiko Yasuda, Sachiyo Nomura, Hong Sheng Cheng, Nguan Soon Tan

**Affiliations:** ^1^ Lee Kong Chian School of Medicine Clinical Sciences Building Nanyang Technological University Singapore 11 Mandalay Road Singapore 308232 Singapore; ^2^ School of Biological Sciences Nanyang Technological University Singapore 60 Nanyang Drive Singapore 637551 Singapore; ^3^ Department of Gastrointestinal Surgery Graduate School of Medicine The University of Tokyo Tokyo 113‐8654 Japan; ^4^ Department of Gastrointestinal Surgery Nippon Medical School Chiba Hokusoh Hospital Chiba 270‐1694 Japan

**Keywords:** autophagy, epithelial‐mesenchymal transition, oxidative stress, *YWHAG* regulome

## Abstract

Metastasis involves epithelial‐to‐mesenchymal transition (EMT), a process that is regulated by complex gene networks, where their deliberate disruption may yield a promising outcome. However, little is known about mechanisms that coordinate these metastasis‐associated networks. To address this gap, hub genes with broad engagement across various human cancers by analyzing the transcriptomes of different cancer cell types undergoing EMT are identified. The oncogenic signaling adaptor protein tyrosine 3‐monooxygenase/tryptophan 5‐monooxygenase activation protein gamma (*YWHAG)* is ranked top for its clinical relevance and impact. The cellular kinome and transcriptome data are surveyed to construct the regulome of *YWHAG*, revealing stress responses and metabolic processes during cancer EMT. It is demonstrated that a *YWHAG*‐dependent cytoprotective mechanism in the regulome is embedded in EMT‐associated networks to protect cancer cells from oxidative catastrophe through enhanced autophagy during EMT. *YWHAG* deficiency results in a rapid accumulation of reactive oxygen species (ROS), delayed EMT, and cell death. Tumor allografts show that metastasis potential and overall survival time are correlated with the *YWHAG* expression level of cancer cell lines. Metastasized tumors have higher expression of *YWHAG* and autophagy‐related genes than primary tumors. Silencing *YWHAG* diminishes primary tumor volumes, prevents metastasis, and prolongs the median survival period of the mice.

## Graphical abstract

The regulome of an oncogenic adaptor protein, tyrosine 3‐monooxygenase/tryptophan 5‐monooxygenase activation protein gamma (*YWHAG*), is integral to initiate and maintain cancer epithelial‐mesenchymal transition (EMT). By increasing autophagic flux, *YWHAG* protects cancer cells from oxidative catastrophe during EMT. Deliberate disruption of *YWHAG* regulome abolishes EMT, limiting secondary tumor growth effectively. It is a promising novel strategy to curb tumor metastasis.

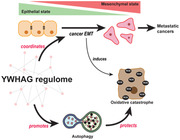

## 1 Introduction

Metastasis is a multistep process resulting in cancer cell dissemination from the primary site to distal organs, along with metabolic adaptation toward prevailing oxidative stress, deprivation of oxygen, and nutrients of the microenvironment at the foreign site. It is often associated with end‐stage cancers.^[^ ^1^ ^]^ Metastasis begins with cancer cells at the primary site undergoing epithelial‐mesenchymal transition (EMT) to breach the basal lamina and invade surrounding tissues, followed by the disruption of adjacent endothelial cells to enter the systemic circulation, evade immune recognition, and survive anoikis before landing on a suitable secondary site. Metastatic cancer cells adapt to the new microenvironment and proliferate to form micrometastases and secondary tumors.^[^ ^2^ ^]^ These metastatic cancer cells are resistant to many chemotherapeutic drugs and other therapeutic modalities, such as chemotherapy and radiotherapy. The complex nature of metastasis makes it a difficult therapeutic target because of its multisystemic spread and enhanced resistance to many anticancer therapies.^[^ ^3^ ^]^ With metastasis accounting for over 90% of cancer mortality,^[^ ^4^ ^]^ effective cancer treatment largely depends on our capacity to intercept the metastatic process. Although these clinical realities have been acknowledged for a long period, our capability to manage metastasis remains unsatisfactorily deficient.

EMT is a series of energy‐demanding events that culminate in the metastatic dissemination of carcinomas.^[^ ^5^ ^]^ During EMT, cancer cells lose epithelial properties and acquire mesenchymal features, including motility and metastatic potential.^[^ ^6^ ^]^ Clinically, active EMT reprogramming often confers an undesirable prognosis in patients due to the acquisition of new characteristics, such as enhanced chemoresistance, invasiveness, and angiogenesis.^[^ ^7^ ^]^ Various signaling pathways, such as transforming growth factor beta (TGF‐β) and NOTCH, and EMT‐inducing growth factors, such as epidermal growth factor (EGF) and platelet‐derived growth factor(PDGF), produced by tumor‐associated stromal cells have been known to confer these oncogenic properties.^[^ ^8^ ^]^ The gene networks that regulate these cellular characteristics are often complex and their activation must be well coordinated to allow functional EMT and metastasis. However, there is little information on the mechanism that coordinates these molecular networks. Deciphering these mechanisms will provide much needed insight into metastasis, where deliberate disruption is expected to yield important clinical information regarding prognosis and response to therapy.

Oncogenic signaling adaptor proteins exert temporal control over activated signaling networks and are prime drivers of the molecular networks coordinating EMT. Unlike adaptor proteins with src homology 2 and phosphotyrosine binding domains, a unique feature of the 14‐3‐3 family of adapter proteins is their ability to interact with serine/threonine phosphorylated target proteins, such as kinases and transcription factors, and modify the activity, stability, and subcellular localization of interacting proteins, hence allowing them to serve as direct regulators of the interacting proteins.^[^ ^9^ ^]^ Thus, the interaction with 14‐3‐3 is tightly regulated by kinases and phosphatases. In mammals, there are seven distinct 14‐3‐3 isoforms (β, γ, ε, η, ζ, σ and τ), each encoded by a different gene. Although the different 14‐3‐3 isoforms were once mistaken to be redundant, increasing studies have illustrated their unique specialized interaction networks.^[^ ^10^ ^]^ The functions and expression of 14‐3‐3 isoforms in cancer cells are different. Among the seven isoforms, 14‐3‐3ζ and 14‐3‐3σ are widely implicated in cancer progression. Elevated 14‐3‐3ζ modulates chemoresistance in diffuse large B‐cell lymphoma and non‐small cell lung cancer, among others.^[^ ^11^ ^]^ A decrease in 14‐3‐3σ expression is reported in primary breast, liver, and stomach tumors.^[^ ^12^ ^]^ The expression of 14‐3‐3γ (also known as tyrosine 3‐monooxygenase/tryptophan 5‐monooxygenase gamma (*YWHAG)* activation protein) was consistently upregulated during EMT in multiple cancer cell lines and in advanced‐stage and human metastatic tumor biopsies.^[^ ^7^, ^13^ ^]^ 14‐3‐3γ also had a larger interactome than other 14‐3‐3 isoforms.^[^ ^14^ ^]^ Taken together, 14‐3‐3 isoforms serve as hubs for many signaling pathways that govern critical processes in cancer.

In this study, we deciphered metastasis/EMT‐associated networks from transcriptomes of cancer cells undergoing EMT, regardless of the inducer. We identified *YWHAG* as a hub gene with broad engagement across the spectrum of human cancers. Using kinomic, transcriptomic, and interactome data, we constructed a *YWHAG* regulome and revealed a cytoprotective mechanism against oxidative stress embedded in EMT‐associated networks.

## 2 Results

### 2.1 Loss of *YWHAG* Delays EMT in Cancer Cells

**Figure 1:**
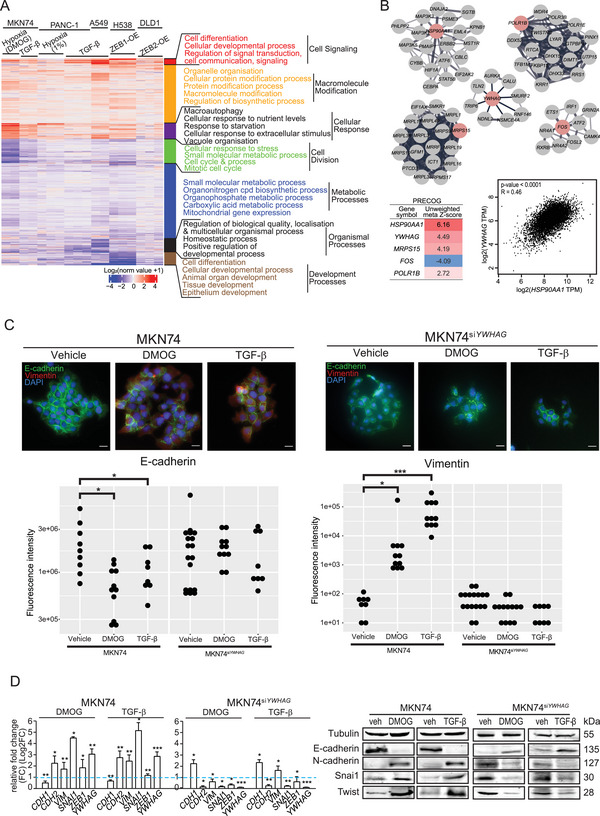
*YWHAG* is a key mediator of EMT during cancer metastasis. A) Heatmap of differential transcriptomes (1270 genes) during EMT induced by different stimuli from 4 publicly accessible and experimentally derived TGF‐β1‐ and DMOG‐induced EMT of MKN74 cells. Red denotes upregulation, while blue denotes downregulation relative to unstimulated control cells. B) Top five hub genes and gene modules (top) involved in cancer cells undergoing EMT and their unweighted meta‐Z scores (bottom left) based on the Prediction of Clinical Outcomes from Genomic Profiles (PRECOG) database. Pearson correlation plot of *YWHAG* and *HSP90AA1* expression in 21 cancer types from The Cancer Genome Atlase (TCGA) database (bottom right). C,D) Representative immunofluorescence images, quantitative polymerse chain reactions (qPCR), and representative immunoblot of epithelial and mesenchymal markers in DMOG (0.5 mm)‐ or TGF‐β (10 ng mL^−1^)‐induced EMT in MKN74 and MKN74^siYWHAG^ cells. In (C), quantifications of the E‐cadherin (green fluorescence) and vimentin (red fluorescence; mesenchymal marker) are shown. In (D), the blue dotted line represents the relative expression of untreated cells. Scale bar = 20 µm. Data are represented as the mean ± SD from at least three independent experiments. ***p < 0.001, **p < 0.01, *p < 0.05; n.s. denotes not significant (Mann‒Whitney U test).

EMT involves transcriptional reprogramming, which often culminates in complex changes to the cellular transcriptome. To identify changes in the EMT transcriptome with broad engagement across the spectrum of human cancers, we interrogated the transcriptomes of four different cancer cell types treated with different EMT inducers (GSE82104, GSE90566, GSE75487, and GSE148823). Semantic similarity and gene ontology enrichment analysis revealed that the 1270 differentially expressed genes (DEGs) can be broadly grouped into seven clusters: cell signaling, cellular response, cell division, metabolic, developmental, and organismal processes, and macromolecular modifications. These functional clusters are ubiquitously altered across different cancer cells during EMT in an inducer‐independent manner, highlighting a functional network that is crucial for successful EMT. These clusters were independently validated using the transcriptomes of MKN74, a polarized gastric carcinoma, undergoing dimethyloxaline glycine (DMOG)‐ and TGF‐β1‐induced EMT (GSE204929) (**Figure** **1**A).

We proceeded to construct gene‐gene interaction networks from the 1270 DEGs using three protein‐protein interaction databases, namely stringDB, Annotation and Integrated Analysis of the 14‐3‐3 interactome (ANIA) and BioPlex databases and identified the hub genes using CytoHubba.^[^ ^14^, ^15^ ^]^ The top five hub genes according to maximal clique centrality scores included heat shock protein 90 alpha family class A member 1 (*HSP90AA1*), RNA Polymerase I Subunit B (*POLR1B*), Mitochondrial Ribosomal Protein S15 (*MRPS15*), Fos proto‐oncogene, AP‐1 transcription factor subunit (*FOS*) and *YWHAG* (Figure 1B). To further understand the relevance of the top five hub genes in cancers, we interrogated the PRECOG (PREdiction of Clinical Outcomes from Genomic profiles) database, which encompasses 166 cancer expression datasets, including overall survival data for ≈18 000 patients diagnosed with 39 distinct malignancies.^[^ ^16^ ^]^ Our analysis revealed that *HSP90AA1* had the highest unweighted meta‐Z score, followed by *YWHAG, MRPS15*, and *POLR1B*. *FOS* had the lowest unweighted meta‐Z score (Figure 1B). The expression of *YWHAG* and *HSP90AA1* was significantly correlated in 21 cancer types from the TCGA database (Figure 1B; Figure S1, Supporting Information). The correlation between *HSP90AA1* and *MRPS15* was *r* = 0.03, while between *YWHAG* and *MRPS15* was *r* = −0.044. Chaperone *HSP90AA1* has previously been shown to promote cancer progression, distant metastasis, and drug resistance.^[^ ^17^ ^]^ However, much less is known about *YWHAG*, an oncogenic adaptor protein in cancer development and progression.

To establish a role for *YWHAG* in EMT, we examined the effect of *YWHAG* suppression on either DMOG‐ or TGF‐β1‐induced EMT in cancer cells. Treatment with both EMT inducers for 48 h increased the cell motility (Figure S2A, Supporting Information), reduced E‐cadherin expression and promoted vimentin staining in the epithelial cell borders (Figure 1C), and overexpressed EMT‐related genes in MKN74, MCF7, and HepG2 cells. *YWHAG* expression was also increased during EMT, regardless of the types of stimuli (Figure 1D). Similar TGF‐β1‐ and DMOG‐induced EMT was also observed in MCF7 and HepG2 cells (Figure S2B,C, Supporting Information). Next, we suppressed *YWHAG* using SMARTpool ON‐TARGETplus siRNA in three cancer cell lines, MKN74^siYWHAG^, MCF7^siYWHAG^, and HepG2^siYWHAG^. Up to ≈80% reduction in the mRNA level of *YWHAG* was observed after 48 h following siRNA transfection (Figure S3A, Supporting Information). Immunoblotting analysis revealed an ≈50% reduction in *YWHAG* protein levels after 8 h of transfection, with minimal reduction in the other six 14‐3‐3 isoforms (β, ε, η, σ, τ, and ζ), confirming the specificity of the *YWHAG* siRNA (Figure S3B, Supporting Information). The suppression of *YWHAG* markedly delayed EMT in cancer cells, as suggested by a reduced Euclidean distance between cells (Figure S2D, Supporting Information) and a reduction in mesenchymal‐associated genes and transcription factors with an increase in epithelial‐associated markers (Figure 1C,D; Figure S2B, Supporting Information). In MKN74 undergoing active EMT and has become mesenchymal‐like. Through the knockdown of *YWHAG* in these mesenchymal‐like MKN74 cells under EMT induction, we observed a stabilization of cell–cell distance and a limitation of cell motility, ceasing further EMT process (Figure S4A–C, Supporting Information). Additionally, the expression of mesenchymal‐associated markers was downregulated, while the expression of E‐cadherin, an epithelial marker, was upregulated (Figure S4D, Supporting Information), suggesting a mesenchymal‐to‐epithelial transition (MET) occurring in these mesenchymal‐like MKN74 cells. When combined with our previous data, these new findings demonstrate the critical role of *YWHAG* in the initiation and maintenance of cancer EMT, and the knockdown of *YWHAG* may promote MET as well.

To ascertain a specific role for *YWHAG* in EMT, we also investigated the effect of *YWHAE* (14‐3‐3ε) and *YWHAH* (14‐3‐3η) knockdown on EMT. *YWHAH* has the highest protein sequence similarity to *YWHAG* and shares the highest overlap in interacting partners (Figure S8A,B, Supporting Information). On the other hand, *YWHAE* has the lowest degree of similarity to *YWHAG*. siRNA knockdown of *YWHAE* and *YWHAH* achieved a reduction of ≈70–80% in the mRNA level of the target genes with minimal reduction in the protein expression of the other six 14‐3‐3 isoforms (Figure S5A–D, Supporting Information). Unlike *YWHAG*‐deficient cancer cells, *YWHAE* or *YWHAH* deficiency did not affect EMT induced by DMOG and TGF‐β. These findings suggest that EMT was not impaired in the absence of either *YWHAH* or *YWHAE* in cancer cells. They also underscore the specific role of *YWHAG* in cancer cells during EMT.

Furthermore, transcriptomic analysis of TGF‐β1‐ and DMOG‐treated wild‐type MKN74 and MKN74^siYWHAG^ revealed that *YWHAG* knockdown markedly activated inflammatory and stress signaling; inhibited cell division, FGF signaling and secondary metabolic processes; and promoted necrotic cell death (Figure S2E, Supporting Information). The results suggest that the loss of *YWHAG* functionality in the presence of EMT inducers is potentially detrimental to cancer cells, highlighting a pivotal role for *YWHAG* in orchestrating many networks involved in EMT‐associated processes.

### 2.2 High *YWHAG* Expression in Tumors is Associated with Poor Prognosis

To ascertain the clinical relevance of *YWHAG*, four cancer databases (Gene Expression database of Normal and Tumour tissues 2 (GENT2), PrognoScan, PRECOG, and TCGA) were interrogated. TCGA database demonstrated that 67.7% (21 out of 31 tumor types) had significantly higher *YWHAG* compared with cognate normal tissues (Figure S6A, Supporting Information), and 22.5% (7 tumor types) and 9.7% (3 tumor types) of tumor types showed no statistically significant difference and an inverse relationship, respectively (Figure S6B, Supporting Information). Likewise, the GENT2 database revealed that 64.7% (22 out of 34) had significantly higher *YWHAG* expression than cognate normal tissues (Figure S6C, Supporting Information), while the remaining 35.3% showed no significant differential expression of *YWHAG* (Figure S6D, Supporting Information). Stratification of the patients into high‐ and low‐*YWHAG*‐expressing tumors based on median expression revealed a poorer prognosis and shorter median survival time in high *YWHAG*‐expressors (Table S1, Supporting Information).

In the PrognoScan cohort database, high *YWHAG* expression increased the risk for distant metastasis (hazard ratio (HR) 1.98‐3.92) as well as relapse or death (HR 1.26‐58.02) (Table S2, Supporting Information). Studies of distant metastasis‐free survival (*n* = 77–87) have reported HRs of 2.28 to 3.92. Comparably, studies of overall survival, relapse‐free survival or disease‐specific survival harmoniously illustrated that patients with high *YWHAG*‐expressing cancers had worse prognoses (HRs of 1.26‐58.02). Likewise, analysis across various cancer types using PRECOG database revealed that *YWHAZ* and *YWHAG* expressed high z scores of 4.16 and 4.13, respectively, while the other well‐established amplified oncogenes, such as *AKT1* and *SRC*, scored 2.55 and 1.95, respectively, where a higher Z score correlates with higher predictive weight in cancer (Table S3, Supporting Information). This global unbiased meta‐analysis indicator strengthened the negative impact of *YWHAG* on the survival of patients diagnosed with different types of cancers.

### 2.3 *YWHAG* Coordinates EMT‐Associated Networks

**Figure 2:**
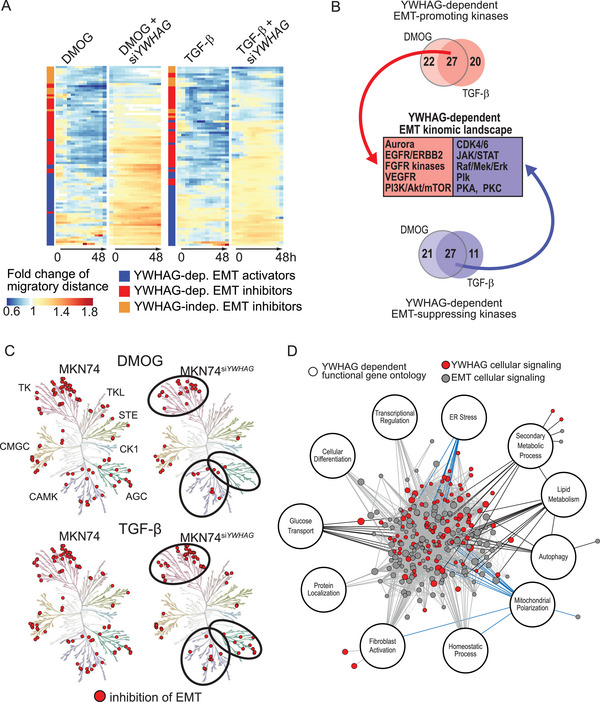
The *YWHAG* regulome coordinates the stress response during EMT. A) Temporal fold changes in migratory distance from 0 to 48 h from the kinase inhibitor screen. EMT was induced in MKN74 and MKN74^siYWHAG^ cells using DMOG (0.5 mm) or TGF‐β (10 ng mL^−1^) and simultaneously challenged with small‐molecule kinase inhibitors. Kinase inhibitors that reduced migratory distance or showed different EMT trends between MKN74 and MKN74^siYWHAG^ were shortlisted and classified into *YWHAG*‐dependent EMT activators (which act on EMT‐attenuating kinases) and *YWHAG*‐dependent/independent EMT inhibitors (which act on EMT‐promoting kinases). B,C) Key *YWHAG*‐dependent protein kinases that coordinate EMT and changes in the EMT kinomic landscape derived from common EMT‐promoting and EMT‐attenuating kinases of DMOG and TGF‐β kinase screens. In (C), the circles highlight that fewer inhibitors targeting the kinases from the tyrosine kinase, AGC and CAMK families were involved in the inhibition of EMT in MKN74^siYWHAG^ compared to MKN74. D) EMT interactome derived from public and in‐house RNAseq databases of different cancers undergoing EMT. Red nodes are *YWHAG*‐mediated cellular signaling pathways that are highly intertwined with other EMT signaling pathways. The *YWHAG*‐dependent functional gene ontologies are annotated. Gene ontologies linked to stress response and cellular metabolism are highlighted with blue and black edges, respectively.

Kinases are essential for the signal transduction of EMT‐associated networks. We surveyed the cellular kinome of MKN74 and MKN74^siYWHAG^ cells using unbiased protein kinase inhibitor screening and live imaging (Figure S7A, Supporting Information). The migratory distance was quantified, and the kinetics of inhibition were shown in heatmaps (**Figure** **2**A; Figure S7B, Supporting Information). Small‐molecule inhibitors that affect EMT can be functionally categorized into EMT inhibitors and activators. During DMOG‐ or TGF‐β1‐induced EMT, EMT inhibitors act on EMT‐promoting kinases to reduce the cell scattering distance, but this effect is lost in *YWHAG*‐dependent kinases following *YWHAG* knockdown (hence, termed *YWHAG*‐dependent EMT‐promoting kinases). In contrast, EMT activators are small‐molecule inhibitors that act on EMT‐attenuating kinases. In the presence of DMOG and TGF‐β1, these kinases were suppressed to enable EMT, so no further change in cell scattering was expected from the EMT activators. In *YWHAG*‐dependent EMT‐attenuating kinases, *YWHAG* knockdown restored their suppressive effect on EMT and susceptibility to EMT activators, resulting in increased cell migratory distance (Figure 2A). Based on the above rationale, we found that most of the small‐molecule inhibitors induced migratory pattern changes following *YWHAG* knockdown (Figure 2A; see Supporting Information for more details). To identify the *YWHAG*‐dependent kinases associated with EMT, we then mapped the small‐molecule inhibitors conferring changing migratory patterns upon *YWHAG* knockdown to their corresponding molecular targets. Comparing DMOG‐ and TGF‐β1‐induced EMT in MKN74 and MKN74^siYWHAG^ cells, a total of 54 inhibitors were identified, with 27 promoting and 27 inhibiting *YWHAG*‐dependent EMT (Figure 2B; Figure S7C, Supporting Information). The kinases predominantly belong to the tyrosine kinase (TK) which is well implicated in cell proliferation and differentiation, and AGC and Ca^2+^/calmodulin‐stimulated protein kinase I (CAMKI) kinase families which are key mediators of metabolism and cellular response (Figure 2C). Upon *YWHAG* knockdown, there was evident loss of kinase activities in kinase families such as the tyrosine kinase‐like (TKL) and CAMKI families. These observations confirm that EMT requires contributions from a multitude of cellular signaling pathways and that most of these pathways are coordinated by *YWHAG*.

To elucidate the interactomes of 14‐3‐3γ (encoded by *YWHAG*), we mined the BioPlex Interactome database, which consists of profiles of protein–protein interactions in nontumorigenic and tumorigenic cell lines. We first identified the number of interacting proteins of the different 14‐3‐3 isoforms. As expected, 14‐3‐3γ had the most interacting proteins at 607, followed by 14‐3‐3η with 561 interacting proteins, while 14‐3‐3ε and 14‐3‐3σ scored the lowest, with 77 and 60 interacting proteins, respectively (Figure S8A, Supporting Information). We also found that 14‐3‐3η, also known as *YWHAH*, has the highest number of overlapping interacting proteins (322 out of 561) with 14‐3‐3γ, despite its weak correlation with cancer, while 14‐3‐3σ (*YWHAS* or stratifin (*SFN)*), which has been associated with cancer drug resistance, has the lowest number of common interacting partners with 14‐3‐3γ (5 out of 60) (Figure S8B, Supporting Information). Functional analysis of the 607 14‐3‐3γ interacting proteins revealed an enrichment in signal transduction, consisting of phosphate‐containing compound metabolic processes and regulation of intracellular signal transduction (Figure S8C, Supporting Information).

To map the regulome of *YWHAG* that coordinates EMT processes, we integrated the *YWHAG*‐dependent kinomic, transcriptomic landscapes and interactomes to construct a *YWHAG* regulome. The regulome revealed a highly intertwined entanglement between *YWHAG*‐mediated signaling and other EMT signaling pathways (Figure 2D). The *YWHAG* regulome highlighted major gene ontologies in cellular metabolism, such as lipid metabolism, autophagy, and glucose transport and stress response, which included endoplasmic reticulum (ER) stress and mitochondrial polarization. These highly intertwined networks highlight *YWHAG* as a signaling hub that coordinates critical processes involved in EMT. Conceivably, *YWHAG* deficiency can perturb transcriptomic reprogramming networks during EMT and may be a novel strategy to curtail metastasis.

### 2.4 *YWHAG* Protects Metastatic Cancer Cells Against Oxidative Cell Death

**Figure 3:**
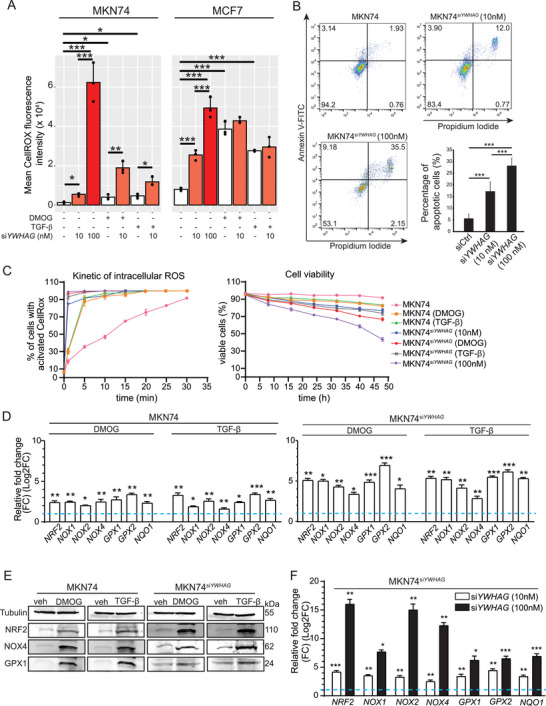
*YWHAG* deficiency induces oxidative stress in MKN74 cells. A) Intracellular ROS levels of MKN74 (left) and MCF7 (right) based on CellROX fluorescence intensity. The cells were transfected with different concentrations of si*YWHAG* (0, 10, and 100 nm) and challenged with EMT inducers like DMOG‐ and TGF‐β. Data from experimental replicates are shown as individual dots in the bar plot. B) AnnexinV‐Propidium iodide FACS profiles of MKN74, MKN74^siYWHAG^ (10 and 100 nm). The percentages of apoptotic cells (Propidium iodide^+^ Annexin V^+^) are summarized in a bar plot. C) Graph showing the kinetics of intracellular ROS (left) and cell viability (right) in untreated, DMOG‐ and TGF‐β‐treated MKN74, MKN74^siYWHAG^, MKN74^siYWHAG^ 10 nm, and MKN74^siYWHAG^ 100 nm cells over a period of 48 h at 8 h intervals. D,E) Relative mRNA and protein expression of oxidative stress markers in MKN74 cells treated with DMOG and TGF‐β. TATA‐box binding protein (*TBP)* and *18S* were used as housekeeping genes for qPCR. β‐Tubulin served as a loading and transfer control for immunoblots from the same samples. F) Relative mRNA expression of oxidative stress markers in MKN74^siYWHAG^ 10 nm and MKN74^siYWHAG^ 100 nm cells. In (D) and (F), the blue dotted line represents the relative expression level from untreated cells. Data are represented as the mean ± SD from at least three independent experiments. ***p < 0.001, **p < 0.01, *p < 0.05; n.s. denotes not significant (Mann‒Whitney U test).

The enhanced cellular metabolism, mitochondrial polarization, and ER stress in the *YWHAG* regulome are suggestive of elevated oxidative stress in cancer cells undergoing EMT.^[^ ^18^ ^]^ We measured higher mean intracellular reactive oxygen species (ROS) in MKN74 cells during DMOG‐ and TGF‐β1‐induced EMT than in untreated MKN74 cells (**Figure** **3**A; Figure S9A, Supporting Information). Kinetic monitoring revealed that ≈20% of MKN74 cells were fluorescent with activated CellRox at the 1st min with a steady increase to 90% at 30th min. Upon exposure to DMOG or TGF‐β1, ≈30% of MKN74 had activated CellRox at the 1^st^ min, which drastically increased to over 80% by 5^th^ min and plateaued at 100% until the end of the examined period (Figure 3C). Silencing *YWHAG* further increased the mean intracellular ROS and the percentage of apoptotic cells in a dose‐dependent manner (Figure 3A,B). Elevated intracellular ROS was also observed in MCF7 cells (Figure 3A; Figure S9A,B, Supporting Information). Increased oxidative stress during EMT occurred in tandem with the overexpression of oxidative stress‐related biomarkers, such as nuclear respiratory factor 2 (NRF2), nicotinamide adenine dinucleotide phosphate oxidase 4 (NOX4) and glutathione peroxidase 1 (GPX1), in wild‐type and *YWHAG*‐knockdown cancer cells during EMT compared with untreated cells (Figure 3C–E; Figure S9C–E, Supporting Information). In contrast, *YWHAG* knockdown had little effect on the viability and apoptosis of noncancerous keratinocytes, HaCaT (Figure S10A–D, Supporting Information), highlighting an oncogenic‐specific role of *YWHAG*. These observations indicate that cancer cells undergoing EMT rapidly accumulate intracellular ROS. *YWHAG* deficiency exacerbated the effect of excessive oxidative stress on reduced cell viability, suggesting a *YWHAG*‐dependent cytoprotective mechanism against oxidative catastrophe during EMT in cancer cells, which is an energy‐demanding process.

### 2.5 *YWHAG* Enhances Autophagic Flux as a Cytoprotective Mechanism

**Figure 4:**
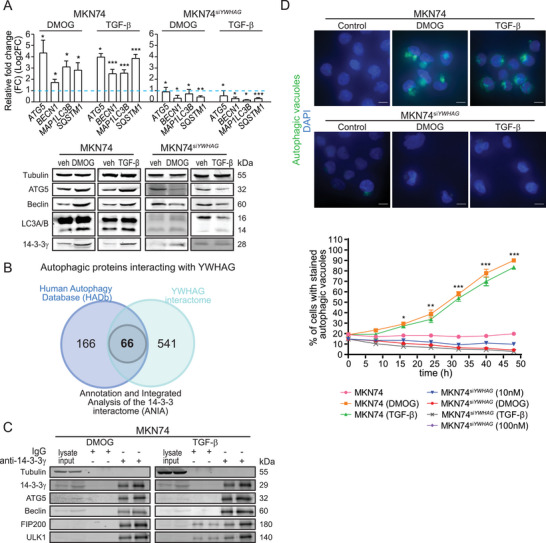
*YWHAG* induces autophagy as a cytoprotective mechanism. A) Relative fold change in mRNA levels (top) and immunoblot analysis (bottom) of autophagy markers (ATG5, Beclin, LC3, sequestosome 1(SQSTM1)) in MKN74 cells treated with DMOG and TGF‐β. The blue dotted line represents the relative expression of untreated cells. *TBP* was used as a housekeeping gene for qPCR, while β‐tubulin was used as a loading and transfer control for immunoblot analysis. B) Number of potential autophagy‐related proteins interacting with *YWHAG*. Combined data from Autophagy‐dedicated Database (HADb), Bioplex Network and ANnotation and Integrated Analysis of the 14‐3‐3 interactome (ANIA) databases. C) Representative immunoblots of autophagy markers (ATG5, Beclin, focal adhesion kinase interacting protein 200 (FIP200) and ULK1) after coimmunoprecipitation with 14‐3‐3γ using MKN74 cells treated with DMOG and TGF‐β. D) Representative images of autophagic vacuoles in DMOG‐ and TGF‐β‐treated MKN74 and MKN74^siYWHAG^ cells to monitor autophagic flux at 48 h. The autophagy dye reagent emits bright fluorescence upon incorporation into preautophagosomes, autophagosomes, and autolysosomes (autophagolysosomes), with minimal staining of lysosomes. The graph illustrates autophagic flux over a period of 48 h at 8 h intervals with a decrease in autophagic flux after *YWHAG* knockdown. Data are represented as the mean ± SD from at least three independent experiments. ***p < 0.001, **p < 0.01, *p < 0.05; n.s. denotes not significant (Mann‒Whitney U test).

Wild‐type cancer cells during EMT did not undergo apoptosis despite the elevated intracellular ROS, unlike their *YWHAG*‐knockdown counterparts, suggesting a cytoprotective mechanism embedded in the EMT‐associated network. Based on our *YWHAG* regulome, the underlying mechanism may involve *YWHAG*‐modulated autophagy. Indeed, overexpression of autophagic proteins (autophagy related protein 5 (ATG5), Beclin and light chain 3 alpha/beta (LC3A/B)) in three cancer cell lines was detected during EMT, which was diminished in *YWHAG*‐knockdown cells (**Figure** **4**A; Figures S11A and S12A, Supporting Information). As an oncogenic adaptor, *YWHAG* could also interact with components of macroautophagy to increase autophagic flux. Through the Human Autophagy‐dedicated Database (HADb), we identified 232 genes that are involved in autophagy and overlapped them with *YWHAG*‐interacting partners (607 proteins) from our earlier interactome analysis. We further identified the *YWHAG*‐interacting autophagic proteins using ANIA, a 14‐3‐3 interactome database. A total of 66 autophagic proteins were identified by high‐throughput 14‐3‐3 affinity capture assays to interact with *YWHAG* (Figure 4B). The top candidates were validated with a coimmunoprecipitation assay using antibodies against 14‐3‐3γ, which showed that 14‐3‐3γ interacted with macroautophagy members, such as Unc‐51 autophagy activating kinase 1 (ULK1), ATG5 and Beclin, in DMOG‐ and TGF‐β‐treated MKN74 cells compared to untreated MKN74 cells (Figure 4C). Approximately 90% of MKN74 cells were positively stained for autophagic vacuoles, including preautophagosomes, autophagosomes and autolysosomes, when challenged with DMOG or TGF‐β, compared to 20% in untreated cells, suggesting that autophagy might be crucial during EMT to mitigate intracellular oxidative damage (Figure 4D). *YWHAG* suppression significantly reduced the number of MKN74^siYWHAG^ cells with autophagic vacuoles compared with MKN74 cells (Figure 4D). The absence of *YWHAG* resulted in a decline in autophagic flux during EMT.

**Figure 5:**
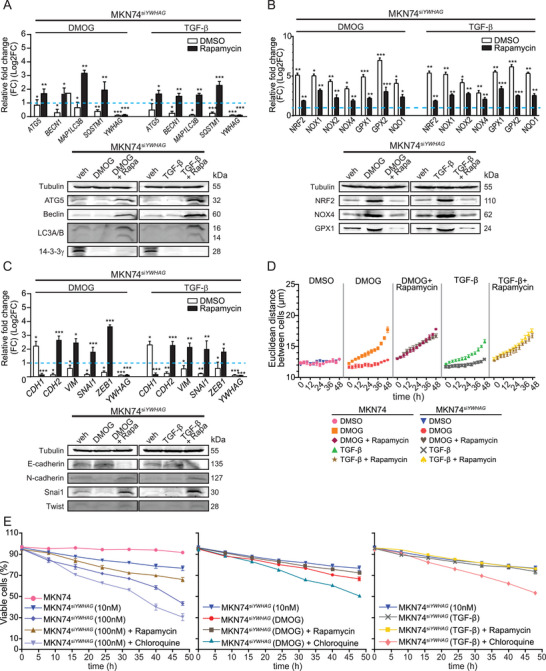
Rapamycin‐induced autophagy rescued EMT arrest caused by *YWHAG* knockdown. A–C) Relative fold change in the mRNA (top) and protein (bottom) levels of autophagic, oxidative, and EMT markers and *YWHAG* in untreated and rapamycin‐treated MKN74^siYWHAG^ cells during DMOG‐ or TGF‐β‐induced EMT. The blue dotted line represents the relative expression level from untreated cells. *TBP* was used as a housekeeping gene for qPCR, while β‐tubulin was used as a loading and transfer control for immunoblot analysis. D) Cell‒cell Euclidean distance of MKN74 and MKN74^siYWHAG^ cells challenged with rapamycin during DMOG‐ or TGF‐β‐induced EMT. E) Percentage of viable MKN74 and MKN74^siYWHAG^ cells given rapamycin or chloroquine during DMOG‐ or TGF‐β‐induced EMT over 48 h at 8 h intervals. Data are represented as the mean ± SD from at least three independent experiments. ***p < 0.001, **p < 0.01, *p < 0.05; n.s. denotes not significant (Mann‒Whitney U test).

Our earlier observations suggest that *YWHAG*‐enhanced autophagy may protect cancer cells undergoing EMT from catastrophic levels of oxidative stress. Conceivably, the deliberate activation of autophagy in *YWHAG*‐knockdown cancer cells during EMT would reduce cell death. By treating MKN74^siYWHAG^, MCF7^siYWHAG^, and HepG2^siYWHAG^ with rapamycin, a potent autophagy inducer, we detected significant upregulation of autophagic markers (**Figure** **5**A; Figures S11B and S12B, Supporting Information). Rapamycin also diminished the deleterious effect of oxidative catastrophe (Figure 5B; Figure S11C, Supporting Information) and restored EMT (Figure 5C,D) and viability (Figure 5E) in MKN74^siYWHAG^ during EMT. Conversely, chloroquine, an inhibitor of autophagy, exacerbated the cytotoxic effect of *YWHAG* knockdown in MKN74 with and without EMT inducers (Figure 5E). Similar effects of rapamycin and chloroquine were also observed in MCF7^siYWHAG^ and HepG2^siYWHAG^ cells undergoing EMT (Figures S11D–F and S12C–E, Supporting Information). Taken together, these observations suggest that *YWHAG* enhances autophagy to lessen oxidative stress‐related damage and improve the survival of cancer cells undergoing EMT.

### 2.6 *YWHAG* Deficiency Attenuates Primary Tumor Growth and Inhibits Metastasis

**Figure 6:**
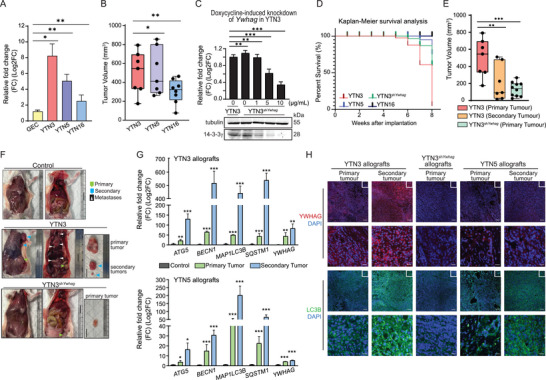
Elevated *YWHAG* expression and autophagy enhance metastasis. A) Relative *YWHAG* mRNA levels in YTN3, 5 and 16 cell lines. B) Volume of primary tumors derived from implanted YTN3, 5 and 16 cancer cells. Primary allograft tumors were measured with a pair of Vernier calipers. The tumor volume was calculated by 0.5× (Length×Width^2^). C) Relative *YWHAG* mRNA (top) and protein (bottom) expression in YTN3^sh^ *^YWHAG^* cells at the indicated doxycycline concentrations. *TBP* was used as a housekeeping gene for qPCR, while β‐tubulin was used as a loading and transfer control for immunoblot analysis from the same samples. D) Kaplan–Meier survival analysis of mice implanted with YTN3, 5, 16 and YTN3^shYWHAG^ cancer cells. E) Volume of primary and secondary tumors derived from implanted YTN3 and YTN3^shYWHAG^ cells. F) Representative images of control mice and mice bearing primary and/or secondary tumors from YTN3 or YTN3^shYWHAG^ cells. G) Relative fold change in the mRNA levels of autophagy markers and *YWHAG* in primary and secondary tumors derived from YTN3 (top) and YTN5 (bottom) cancer cells. H) Representative images of immunofluorescence staining of *YWHAG* and LC3B in tissue sections from YTN3, YTN3^shYWHAG^ and YTN5 allografts. Scale bar (first and third panels) = 100 µm. Scale bar (second and fourth panels) = 20 µm. Data are represented as the mean ± SD from at least three independent experiments. *n* = 10 mice/treatment group. ***p < 0.001, **p < 0.01, *p < 0.05; n.s. denotes not significant (Mann‒Whitney U test).

To confirm an in vivo role for *YWHAG*, we first investigated the expression of *YWHAG* in N‐methyl‐N‐nitrosourea‐induced murine glandular stomach carcinoma cell lines, namely, YTN3, YTN5 and YTN16.^[^ ^19^ ^]^ *YWHAG* was highly expressed in YTN3, followed by YTN5 and YTN16, when compared to gastric epithelial cells obtained from NSG mice (**Figure** **6**A). Tumors derived from these three YTN cancer cell lines were analyzed at 8 weeks post implantation. Mice injected with YTN3 cells displayed the largest tumor volumes, followed by YTN5 and YTN16 cells (Figure 6B). We also generated a transgenic YTN3^shYWHAG^ cell line that carries a doxycycline‐inducible vector for sh*YWHAG* to achieve in vivo *YWHAG* suppression (Figure 6C). YTN3^shYWHAG^ allografted mice were fed a doxycycline diet only from the second week onward to suppress the expression of *YWHAG*, as premature inhibition of *YWHAG* resulted in the absence of the allografted tumor. YTN5 and YTN16 allograft models survived until the end of experiments at the 8th week. On the other hand, ≈90% of YTN3 allografted mice did not survive beyond eight weeks (Figure 6D). Mice harboring YTN3‐derived tumors presented with spontaneous intraperitoneal hemorrhage and splenomegaly at the time of death. Further examinations revealed numerous metastasized tumors to distant sites, such as the liver, thoracic cavity, and intraperitoneal region (Figure 6E,F). In contrast, ≈70% of the mice survived until the end of the experiments with doxycycline‐induced sh*YWHAG*, and YTN3^shYWHAG^‐derived primary tumors were smaller than YTN3 tumors (Figure 6D,E). Only one YTN3^shYWHAG^ allografted mouse had a secondary tumor. Consistent with our in vitro findings, secondary tumors from YTN3 and YTN5 allografts showed significantly higher levels of *YWHAG* and autophagy‐related genes than primary tumors (Figure 6G).

To validate our autophagy flux and *YWHAG* findings in our in vitro models, tumor sections from YTN3, YTN3^sh^ *^YWHAG^* and YTN5 allografts were stained for the autophagic markers LC3B and 14‐3‐3γ. Increased 14‐3‐3γ staining was observed in the metastasized tumors when compared with YTN3‐ and YTN5‐derived primary tumors. Both the primary and metastasized tumors from YTN3 allografts expressed higher levels of *YWHAG* than those from YTN5 allografts. Reduced 14‐3‐3γ staining was observed in YTN3^shYWHAG^ allografts due to the knockdown of *YWHAG*. The LC3B staining profiles mirrored those of *YWHAG* (Figure 6H). Finally, patients’ metastatic tumors had consistently elevated expression of *YWHAG* and autophagic proteins compared with lower‐grade tumors (Figure S13, Supporting Information). Together, these observations suggest that *YWHAG* contributes to the growth and metastatic potential of the primary tumor and reduces the overall median survival time. In addition, *YWHAG* expression and autophagy are correlated with cancer progression in a stage‐dependent manner regardless of cancer type and model.

## 3 Discussion

In this study, we showed that the regulome of the oncogenic signaling adaptor *YWHAG* is a key constituent of EMT and metastasis mechanisms with broad clinical impact across different human cancer types. This coordinating function is specific to *YWHAG*, which was not observed for other 14‐3‐3 members, namely, *YWHAE* and *YWHAH*. We constructed a *YWHAG* regulome that is enriched in genes involved in cellular metabolism and the stress response. These highly intertwined networks highlight *YWHAG* as a signaling hub that coordinates EMT‐associated processes via its interaction with a plethora of functionally different proteins, hence coordinating a wide array of regulatory networks. We further demonstrate that a *YWHAG*‐dependent cytoprotective mechanism is embedded in EMT‐associated networks that protect cancer cells from oxidative catastrophe by *YWHAG* interaction with macroautophagic machinery and enhancing autophagy. Finally, *YWHAG* deficiency leads to increased intracellular oxidative stress and impairs autophagy, substantially inhibiting tumor growth and metastasis.

Two key cellular activities emerge when we examine the *YWHAG* regulome during EMT: metabolism and stress response. A previous study showed that *YWHAG* elevates the cellular adenylate energy flux via interaction with tuberous sclerosis complex proteins (TSC) and Snai1.^[^ ^7a^ ^]^ Such interactions reprogram cancer metabolism through increased lipid and glucose catabolism to secure ample cellular energy that fuels EMT and other ancillary activities, i.e., chemoresistance.^[^ ^7^ ^]^ The resultant high ROS generation caused by metabolic overdrive is mitigated by a *YWHAG*‐dependent cytoprotective mechanism through enhanced autophagy, which is both a nutrient recycler and a repair mechanism for irreversibly damaged molecules due to overwhelming oxidative stress.^[^ ^20^ ^]^ *YWHAG* has been implicated in autophagy.^[^ ^21^ ^]^ The phosphorylation of endonuclease G, which is a mitochondrial nuclease, strengthens its binding affinity with *YWHAG*, leading to mammalian target of rapamycin (mTOR) suppression and autophagy initiation. Our data reveal that autophagy is a pivotal process for cancer metastasis, and *YWHAG* deficiency dismantles the EMT‐associated network and ceases these processes. Conceivably, by coordinating autophagy, *YWHAG* offers a prosurvival and cytoprotective mechanism that enables metabolic flexibility, self‐renewal, and repair mechanisms to counteract energy‐demanding EMT and metastatic cascades. Our findings showed the significant influence that *YWHAG* has on metabolic and transcriptional reprogramming in metastasis.

Our comprehensive analysis by interrogating the TCGA and GENT2 databases revealed that almost 70% of deposited tumors, mostly solid tumors, displayed significantly higher *YWHAG* expression than cognate normal tissues. This suggests that *YWHAG* is often overexpressed in most solid tumors, such as pancreatic, gastric, lung, breast, and hepatocellular carcinoma, and that *YWHAG* overexpression leads to the activation of signaling pathways that drive cancer progression.^[^ ^7^, ^22^ ^]^ High *YWHAG* expression correlates with poor prognosis in patients. By stratifying patients into high‐ and low‐*YWHAG* expressing tumors, we observed that patients with high‐*YWHAG* expressing tumors were found to have an overall shorter median survival time, reemphasizing the clinical relevance of *YWHAG* in metastasis and disease relapse.

While comprehensive molecular characterization of human cancers has advanced cancer diagnostics and treatments, a large and untapped therapeutic space is occupied by nonenzymatic proteins that can be targeted only through their regulome. *YWHAG*, an oncogenic signaling adaptor protein, is a highly promising target against tumor metastasis. Thus, network perturbations through the disruption of the regulome of *YWHAG* offer a potentially novel and effective strategy to curtail tumor metastasis. *YWHAG* is an atypical target that lacks a ligand‐binding pocket and enzymatic activity; instead, *YWHAG* regulates many signaling pathways via its interactomes, rendering it at the center of an adaptive signaling hub. Currently, there is no small‐molecule inhibitor of *YWHAG*. Therefore, a conventional pharmacotherapy‐based approach may not be applicable to target *YWHAG*. In this context, the US Food and Drug Administration recently approved the first RNA‐based gene silencing drug,^[^ ^23^ ^]^ which paves the way for therapeutically inhibiting other nonenzymatic targets, such as *YWHAG*. Although *YWHAG* deficiency did not affect the viability of nontumorigenic cells, marrying si*YWHAG* with targeted delivery strategies will enhance its safety profile. The *YWHAG* interactome also represents a novel target space for protein‒protein interaction (PPI) modulators in metastatic cancer. There has been increased interest by pharmaceutical companies in developing drugs targeting PPIs in recent years. PPI inhibitors, including BH3 and second mitochondrial‐derived activator of caspase mimetics and tubulin polymerization inhibitors, are often used in anticancer drugs.^[^ ^24^ ^]^ Studies have explored the crystal structures of 14‐3‐3 members, allowing us to better understand the target domain interaction surfaces. The sequence of the target proteins of *YWHAG* has been elucidated to involve the RXXpS/TXP sequence.^[^ ^25^ ^]^ While this approach was not investigated in this study, deliberate disruption of the *YWHAG* interactome will yield important clinical information on metastasis.

## 4 Conclusion

To conclude, we elucidated a *YWHAG* regulome that coordinates gene regulatory networks for EMT‐associated processes in metastatic cancer cells. We further revealed that the regulome involves a cytoprotective mechanism that protects metastatic cancer cells from oxidative cell death by modulating autophagy. Disruption of the *YWHAG* regulome offers a new strategy to curtail tumor metastasis.

## 5 Experimental Section

### Cell Culture

The polarized human gastric adenocarcinoma cell line MKN74 and the human epidermal keratinocyte cell line HaCaT were cultured as previously described.^[^ ^7^, ^26^ ^]^ YTN3, YTN5, and YTN16, *N‐methyl‐N‐nitrosourea*‐induced murine glandular stomach carcinoma cells, were kind gifts from Prof. Tetsuya Tsukamoto (Fujita Health University, Aichi, Japan).^[^ ^19^ ^]^ Human breast carcinoma, MCF7 and hepatocellular carcinoma, HepG2, were purchased from American Type Culture Collection and cultured in RPMI‐1640 and DMEM supplemented with 10% FBS, respectively.

### In Vitro EMT Models

TGF‐β1 (10 ng mL^−1^) (Peprotech, New Jersey, USA) or dimethyloxaline glycine (DMOG) (0.5 mm) (Sigma–Aldrich, Missouri, USA) was used to induce EMT. Cells were seeded at 525 cells cm^−2^ in 10 cm dishes 2 days before the experiment. The culture medium was replaced with serum‐free medium prior to the addition of EMT inducers. Cell viability was determined with trypan blue. MKN74^siYWHAG^ cells were transfected with 10 nm siRNA for 8 h before the medium was replaced with complete RPMI‐1640 for an hour before replacing with serum‐free medium containing EMT inducers for 48 h. Autophagy was triggered with 1 nm rapamycin (Sigma–Aldrich, Missouri, USA) and inhibited using 50 µm chloroquine (Sigma–Aldrich, USA) for 48 h.

### Generation of the Inducible YTN3^shYWHAG^ Cell Line

A doxycycline‐inducible pSingle‐tTS‐shRNA vector carrying sh*YWHAG* was transfected into YTN3 cells using Lipofectamine 2000. The murine *YWHAG* shRNA target sequence was 5′‐ CTCGAGGGACAACTACCTGATCAAGAAAAGCTT‐3′. YTN3^shYWHAG^ cells were selected with 500 µg mL^−1^ G418. YTN3^shYWHAG^ cells were treated with doxycycline (1–10 mg mL^−1^) (Sigma–Aldrich, USA) before being harvested. The knockdown efficiency was verified by qPCR and immunoblotting.

### Transcriptomic Meta‐Analysis

A repository search was conducted up to 2021 from databases to identify RNA‐seq studies for in vitro EMT models using cancer cell lines. Four RNA‐Seq datasets (GSE82104, GSE90566, GSE75487, and GSE148823) were downloaded from the repository, of which one experiment treated PANC1 and A549 with TGF‐β1 (GSE90566), another one induced hypoxia in PANC1 with cobalt(II) chloride (CoCl2) (GSE82104), and two overexpressed Zinc Finger E‐Box Binding Homeobox 2 (Zeb1) in H538 (GSE75487) and Zinc Finger E‐Box Binding Homeobox 1 (Zeb2) in DLD1 (GSE148823), respectively. The in‐house sequencing data of MKN74 cells with EMT induction have been deposited in the GEO database (GSE204929).

DESeq2 was used to perform differential gene expression between the control (no EMT) and treatment groups of the aforementioned datasets separately.^[^ ^27^ ^]^ To integrate the datasets, we selected genes which were either consistently up‐ or downregulated in at least 80% of the datasets. One thousand two hundred seventy genes representing the common EMT‐associated transcriptomic profile across five cancer cell types and six EMT inducers were shortlisted, of which 494 genes were upregulated and 776 genes were downregulated. Functional enrichment analysis of the EMT‐associated genes was carried out using ViSEAGO.^[^ ^28^ ^]^ Using a semantic similarity method of best‐matched average in ViSEAGO, the DEGs were clustered into seven main functional clusters.

### Kinase Inhibitor Array

MKN74 and *YWHAG*‐knockdown MKN74 (MKN74^siYWHAG^) cells were treated with two kinase inhibitor panels (SYN‐2103; SYNkinase, Victoria, Australia & TargetMol, Massachusetts, USA) comprising 188 distinct small‐molecule inhibitors collectively targeting 130 unique protein kinases^[^ ^29^ ^]^ in the presence of EMT inducers. Treated cells were imaged over 48 h using JuLi Stage (NanoEnTek Inc., Seoul, Korea). The Euclidean distance between multivariate centroids of the cell populations was considered the migratory distance and measured at 4 h intervals for 48 h. The log fold change of the migratory distance of cancer cells treated with kinase inhibitors compared to untreated cells was determined. The temporal changes in the migratory distance were expressed as the area under the curve (AUC) and compared between wild‐type MKN74 and MKN74^siYWHAG^ to identify protein kinases implicated in EMT. *YWHAG*‐dependent/*YWHAG*‐independent EMT inhibitors were categorized using the quantile‐based cutoffs according to the following criteria (Figure S6, Supporting Information):

1) Kinase inhibitors with AUCs ≤ 25th percentile in MKN74 and MKN74^siYWHAG^ are classified as **YWHAG‐independent EMT inhibitors**
2) Kinase inhibitors with AUCs ≤ 25th percentile in MKN74, but > 25th percentile in MKN74^siYWHAG^ are identified as **YWHAG‐dependent EMT inhibitors**
3) Kinase inhibitors with AUCs < 75th percentile in MKN74, but ≥ 75th percentile in MKN74^siYWHAG^ are labeled as **YWHAG‐dependent EMT activators**

KinMap was used to illustrate the kinomic changes in *YWHAG*‐knockdown MKN74 cells during EMT (Figure 2C).^[^ ^30^ ^]^

### Construction of *YWHAG* Regulome During EMT

Data integration between EMT and *YWHAG*‐dependent biological activities was performed at the functional level based on a published workflow with some modifications.^[^ ^31^ ^]^ Functional enrichment analyses of the EMT‐associated DEGs (1270 genes; Figure 1A), *YWHAG*‐dependent kinases, and DEGs (Figure 2B; Figure S2E, Supporting Information) were carried out using ViSEAGO^[^ ^28^ ^]^ to identify EMT‐ and *YWHAG*‐associated functional changes. Enriched gene functions associated to EMT and *YWHAG* with a false discovery rate (FDR) < 0.05 were loaded to Cytoscape to construct a connectivity network based on the number of shared genes between the functions (edge cutoff similarity = 0.375).^[^ ^32^ ^]^ The nodes (which are the enriched functions) were color‐coded to reflect their origins (EMT‐ or *YWHAG*‐associated functional changes). The nodes were grouped using AutoAnnotate plugins to annotate the functional clusters. Compound Spring Embedded (CoSE) Cluster layout was used to construct the layout of the network and segregate the functional clusters.

### Immunofluorescence and Immunohistochemistry

EMT was identified by immunofluorescence staining for E‐cadherin and vimentin as previously described.^[^ ^7a^ ^]^ The primary and secondary antibodies used are listed in Table S4 (Supporting Information). Cells were counterstained with DAPI and visualized with Carl Zeiss Axio Observer 7 (Carl Zeiss, Germany). Tumor biopsies were fixed, dehydrated, paraffin embedded, and processed into tumor sections as previously described.^[^ ^33^ ^]^ Images of the sections were acquired using a Zeiss Axio Scan Z1 (Carl Zeiss, Germany).

### CellROX Green Assay

Following EMT induction, 5 µm CellROX Green reagent (Thermo Fisher Scientific, USA) was added to the cells and incubated at room temperature in the dark. Cells were washed with phosphate buffered saline (PBS) twice before resuspension in PBS for flow cytometry using BD Accuri C6 Plus (BD Biosciences, USA).

### Autophagic Flux Assay

The presence of preautophagosomes, autophagosomes, and autolysosomes (autophagolysosomes) in MKN74 and MKN74^siYWHAG^ cells during EMT was determined using Autophagy Assay kit according to the manufacturer's instructions (ab139484, Abcam, UK). Images were acquired at 40× immediately after staining with 488 nm excitation using Carl Zeiss Axio Observer 7 (Carl Zeiss, Germany).

### In Vivo Induction of EMT

Eight weeks old NSG mice (18–21 g) were randomized into different cages and subcutaneously injected with 1 × 10^6^ of YTN5, YTN16, YTN3, or YTN3^shYWHAG^ cells resuspended in Matrigel, respectively. All YTN3^shYWHAG^ mice were fed a doxycycline diet (625 mg kg^−1^) (Envigo, USA) 2 weeks postinjection. The tumors were monitored and measured with a Vernier caliper every other day. The sample size (*n* = 10 mice/treatment group) was determined using a power analysis. At endpoint, the mice were euthanized with carbon dioxide and primary and secondary tumors were harvested. The tumors were snap frozen in liquid nitrogen and stored at −80 °C until further analysis or fixed in 4% paraformaldehyde for tissue processing. All procedures in the animal experimentation were performed according to the Nanyang Technological University's Institutional Animal Care and Use Committee guidelines (A19032, A19034).

### Statistical Analysis

Statistical analyses were performed with two‐tailed nonparametric Mann–Whitney tests. A *p* value of < 0.05 was considered significant (*p < 0.05; **p < 0.01; ***p < 0.001; n.s., not significant).

### Ethics Approval

All procedures in the animal experimentation were performed according to the Nanyang Technological University's Institutional Animal Care and Use Committee guidelines (A19032, A19034).

## Conflict of Interest

The authors declare no conflict of interest.

## Author Contributions

N.S.T., J.X.T.L., and H.S.C. designed and performed the experiments and interpreted the data. J.X.T.L., W.R.T., Z.S.L., W.D.C.Y., B.S., and H.S.C. performed the meta‐analysis. J.X.T.L. and J.Q.L. performed the kinase inhibitor screen. J.X.T.L. and H.S.C. performed the kinase inhibitor screen analysis. J.X.T.L. and D.C. performed the FACS analysis. T.Y. and S.N. generated the YTN3 cell lines. J.X.T.L. and M.I.G.V. performed the in vivo animal experiments. N.S.T., J.X.T.L, and H.S.C. wrote the manuscript. All authors reviewed the manuscript.

## Supporting information

Supporting Information

Supporting Information File 1

Supporting Information File 2

## Data Availability

The data that support the findings of this study are available in the supplementary material of this article.
