# Corrigendum to “YWHAG Deficiency Disrupts the EMT‐Associated Network to Induce Oxidative Cell Death and Prevent Metastasis”

**DOI:** 10.1002/advs.202506464

**Published:** 2025-05-23

Lee, J. X. T., Tan, W. R., Low, Z. S., Lee, J. Q., Chua, D., Yeo, W. D. C., See, B., Vos, M. I. G., Yasuda, T., Nomura, S., Cheng, H. S., and Tan, N. S. (2023). YWHAG Deficiency Disrupts the EMT‐Associated Network to Induce Oxidative Cell Death and Prevent Metastasis. *Advanced science (Weinheim, Baden‐Wurttemberg, Germany)*, *10*(31), e2301714. https://doi.org/10.1002/advs.202301714

1. In the originally published version of this article, errors were identified in Figure 4C, Figure S2B,C (Supporting Information). The Western blot image of 14‐3‐3γ in Figure 4C for “DMOG‐treated MKN74” was accidentally used for “TGF‐β‐treated MKN74.” In Supplementary Figure S2B, the western blot images of N‐cadherin for “TGF‐β‐treated MCF7” was accidentally used for the “DMOG‐treated MCF7^si^ *^YWHAG^*” sample. In Figure S2C (Supporting Information), the Western blot image of 14‐3‐3γ in Figure S2C (Supporting Information) was inadvertently used for Twist in “DMOG‐treated HepG2.” The correct figures are presented below. The authors state that these corrections do not influence the data analysis or conclusions and that they sincerely apologize for the unintentional errors.


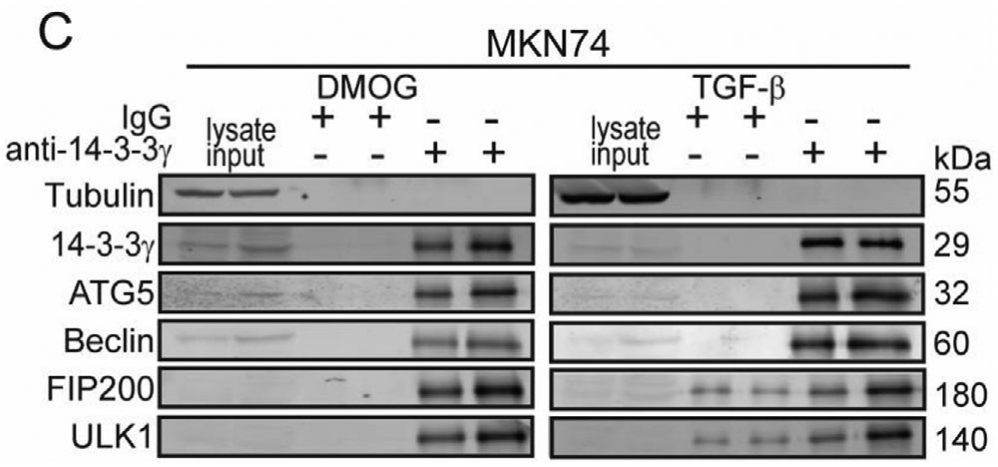


Figure 4C.


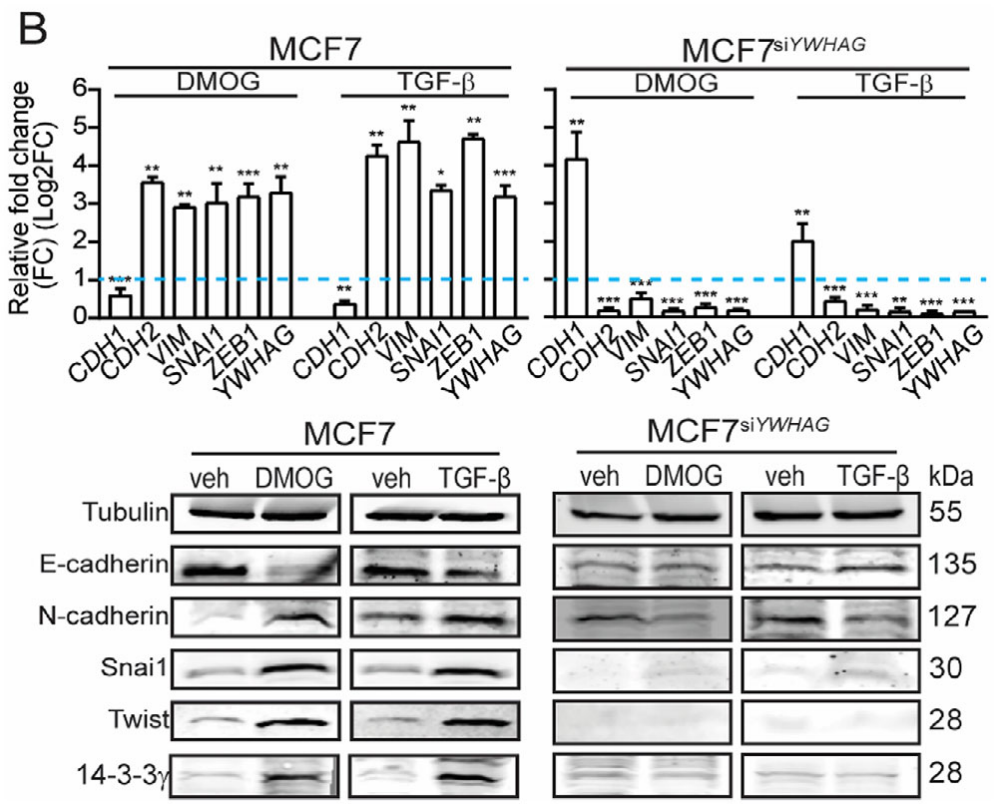


Figure S2B


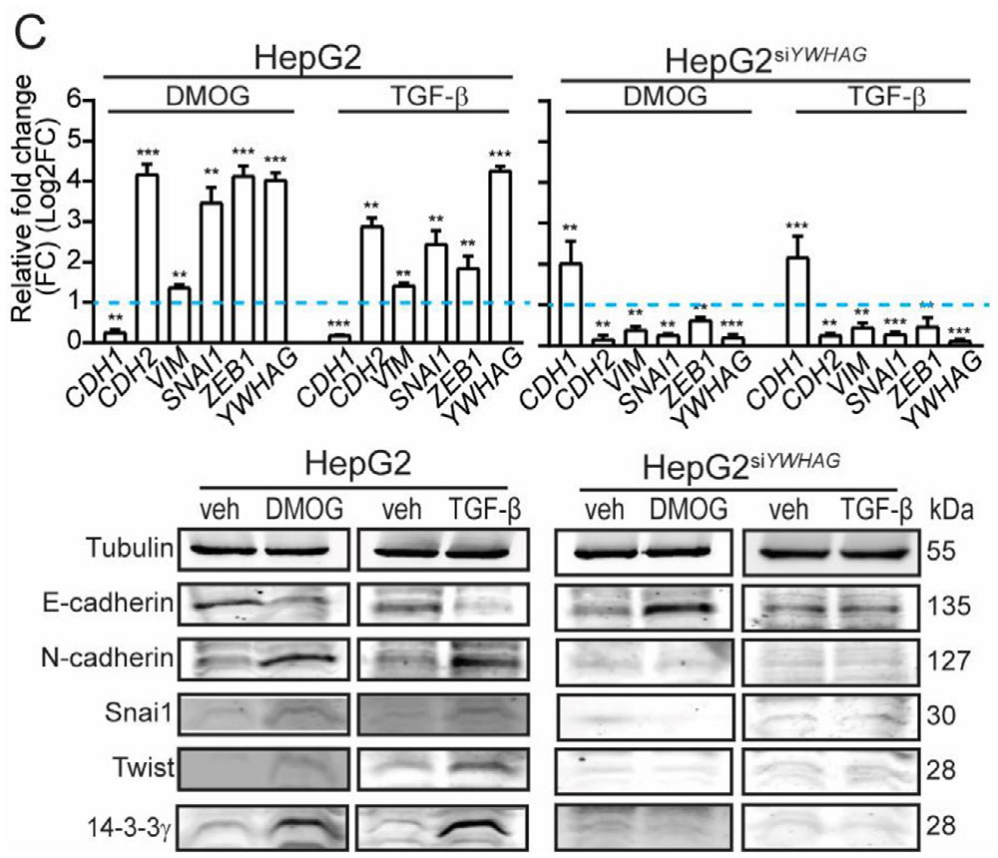


Figure S2C

