# Supplementary material for: YWHAG Deficiency Disrupts the EMT‐Associated Network to Induce Oxidative Cell Death and Prevent Metastasis
Source: Adv Sci (Weinh). 2023 Sep 27;10(31):2301714. doi: 10.1002/advs.202301714 (PMC10625110; doi:10.1002/advs.202301714)
Supplement: Supplementary file 1 — Supporting Information [file ADVS-10-2301714-s001.pdf]

ADVANCED SCIENCE
Open Access

Supporting Information

for *Adv. Sci.*, DOI 10.1002/advs.202301714

YWHAG Deficiency Disrupts the EMT-Associated Network to Induce Oxidative Cell Death and Prevent Metastasis

Jeannie Xue Ting Lee, Wei Ren Tan, Zun Siong Low, Jia Qi Lee, Damien Chua, Wisely Duan Chi Yeo, Benedict See, Marcus Ivan Gerard Vos, Tomohiko Yasuda, Sachiyo Nomura, Hong Sheng Cheng and Nguan Soon Tan**

Supplemental information

YWHAG deficiency disrupts the EMT-associated network to induce oxidative cell death and prevent metastasis.

Jeannie Xue Ting LEE[1], Wei Ren TAN[1], Zun Siong LOW[1], Jia Qi LEE[2], Damien CHUA[1], Wisely Duan Chi YEO[2], Benedict SEE[2], Marcus Ivan Gerard VOS[1], Yun Sheng YIP[1], Tomohiko YASUDA[3,4], Sachiyo NOMURA[3], Hong Sheng CHENG[1 *], Nguan Soon TAN[1,2 *]

[1] Lee Kong Chian School of Medicine, Nanyang Technological University Singapore, Clinical Sciences Building, 11 Mandalay Road, 308232 Singapore, Singapore

[2] School of Biological Sciences, Nanyang Technological University Singapore, 60 Nanyang Drive, 637551 Singapore, Singapore

[3] Department of Gastrointestinal Surgery, Graduate School of Medicine, The University of Tokyo, Tokyo, Japan.

[4] Department of Gastrointestinal Surgery, Nippon Medical School Chiba Hokusoh Hospital, Chiba, Japan.

*Correspondence: HSC (Email: hscheng@ntu.edu.sg), senior corresponding author NST (Email: nstan@ntu.edu.sg; Tel. +65-6904-1295)

Running title: The YWHAG regulome coordinates EMT-associated processes.

Supplemental Material and Methods

Immunoblotting. Cells were lysed using ice-cold RIPA buffer (Sigma Aldrich, USA), and protein was quantified using Braford Assay (Bio-Rad Laboratories, California, USA). Protein extracts were resolved by SDS–PAGE and electrotransferred onto nitrocellulose membranes (Merck & Co., New Jersey, USA). The membranes were blocked in 1X Odyssey Blocking buffer (LI-COR Biosciences, Nebraska, USA) for 1 h at room temperature. The membranes were then incubated with the respective primary antibodies in 1X Odyssey Blocking buffer (**Table S4**) overnight at 4°C. Membranes were washed three times with TBST (1 X TBS, 50 mM Tris-HCl, pH 7.6, 150 mM NaCl, 0.01% Tween-20) and further incubated with the respective IRDye® 680-conjugated or 800-conjugated anti-IgG secondary antibodies (LI-COR Biosciences USA) in 1 X Odyssey Blocking Buffer for 1 h at room temperature. Next, the membranes were washed with TBST with 0.01% SDS before analysis using an ODYSSEY CLx Infrared Imaging system (LI-COR Biosciences, USA).

Immunoprecipitation. Cells were lysed using ice-cold RIPA buffer (Sigma Aldrich, USA). The lysate was then incubated with anti-14-3-3γ antibodies (Abcam, UK) overnight at 4°C with constant rotation. Anti-14-3-3γ antibodies were then affinity precipitated using protein A/G beads (Santa Cruz, California, USA). The cell pellet was washed three times with PBS before resuspension in Lamelli's buffer (Bio-Rad Laboratories, USA). Proteins were released by boiling the samples for 10 min. Immunoblotting analyses were carried out as mentioned above, and the antibodies used are described in **Table S4**.

siRNA knockdown. SMARTpool ON-TARGETplus™ siRNA, which consists of a mixture of four siRNAs targeting *YWHAG*, *YWHAE* and *YWHAH*, was purchased from Dharmacon (Thermo Fisher Scientific, USA). Cancer cells were seeded in 96-well plates at a concentration of 300 cells/well prior to siRNA transfection with Lipofectamine 2000 (Invitrogen, Massachusetts, USA) according to the manufacturer's instructions. The siRNA-treated cells were incubated for 8 h at 37°C before the medium was replaced with complete medium for an hour and further incubated in the respective medium for 48 h.

RNA extraction and real-time PCR. Total RNA was extracted using TRIzol® Reagent (Thermo Fisher Scientific, USA) followed by Pure NA. Fastspin Total RNA Extraction Kit (Research Instruments, USA) according to the manufacturer's protocol. Total RNA was quantified based on the A260/280 absorbance

using a Nanodrop ND1000 (Thermo Fisher Scientific, USA). Total RNA was treated with DNaseI (Thermo Fisher Scientific, USA) and reverse transcribed using qScript cDNA SuperMix (Quantabio, Massachusetts, USA) according to the manufacturer's instructions. Quantitative PCR (qPCR) was performed in triplicate using the KAPA SYBR FAST qPCR master mix (Kapa Biosystems, Massachusetts, USA) and the C1000 Thermal Cycler with CFX96 Real-Time System module (Bio-Rad Laboratories, USA). Human and mouse primer sequences are listed in **Table S5**. Please refer to **Supporting File 2** for the MIQE checklist for the reporting of our qPCR experiments.

FACS Apoptosis Assay. Cells were detached from the plates before washing with ice-cold 1x PBS twice followed by resuspension in 1x binding buffer (BD Biosciences, USA). The cells were stained concurrently with 5 µL of fluorescein isothiocyanate (FITC) Annexin (BD Biosciences) and 5 µL of propidium iodide (PI) (BD Biosciences, USA) for 15 minutes at room temperature. The cells were washed again with ice-cold 1x PBS before resuspending in 400 µL of 1x binding buffer. Flow cytometry was performed using BD Accuri C6 Plus, and analysis was performed using FlowJo X software.

RNA sequencing and bioinformatic analysis. mRNA was sequenced using NovaSeq6000 (Illumina, California, USA) to yield 150-bp paired-end reads. All data were uploaded via puTTy and Filezilla onto the National Supercomputing Centre Singapore (NSCC) (Singapore, Singapore) and subsequently processed. FASTQC (v.0.11.8) was used to assess the read quality. Low-quality reads were trimmed with Trimmomatic (V.0.38). BBmap (BBmap v38.42) and Hisat2 (v2.1.0) were used for read alignment to the GRCh38 human genome. FeatureCounts (v.1.6.3) [1] was used to generate the gene counts. DESeq2 was used to analyse the differential gene expression between the control and treatment groups. Principal component analysis (PCA) was used to visualize the clustering pattern of the datasets. Metabolic genes were retrieved from Gene Ontology, EMT genes were obtained from EMTome, and autophagy genes were obtained from the Human Autophagy Database (HADb). VISEAGO was used to identify and group genes by their biological processes [2]. The *YWHAG*-EMT interactome was constructed using the functional enrichment outputs according to a published method [3]. The sequencing data of MKN74 cells with EMT induction have been deposited in the GEO database (GSE204929). Ingenuity Pathway Analysis (IPA) software (Qiagen, Germany) was employed to carry

out the analysis of pathways and map downstream signalling molecules and possible interactions between 14-3-3γ and proteins involved both directly and indirectly in autophagy [4].

Cytoscape analysis. ClueGo is a plug-in on Cytoscape that combines gene ontology terms and Kyoto Encyclopaedia of Genes and Genomes (KEGG) pathways to allow for an integrated network analysis [5]. ClueGo was employed in this study to cluster the 14-3-3γ interactomes isolated from BioPlex into their respective ontology terms. CytoHubba was utilized to compute the maximal clique centrality (MCC) scores and rank the nodes in the different networks obtained during meta-analysis [6]. Genes with the top 5 highest MCC scores were identified as the hub genes in the EMT interactome.

Databases. Four datasets consisting of different EMT induction methods were retrieved from the Gene Expression Omnibus (GEO) repository. Out of the four datasets obtained, one used TGF-β1 on PANC-1 and A549 (GSE90566), another one induced hypoxia in PANC-1 with $CoCl_2$ (GSE82104) and two overexpressed Zeb1 in H538 (GSE75487) and Zeb2 in DLD-1 (GSE148823).

The Human Autophagy Database (HADb), which is the first Human Autophagy-dedicated Database with information about the human genes involved in autophagy, was used to retrieve a complete and up-to-date list of human proteins involved both directly and indirectly in autophagy as described in the literature.

EMTome is a database that integrates profiles across multiple subtypes of cancers and was selected from The Cancer Genome Atlas (TCGA), which comprises the gene signatures, multiomics features, immune profiles and interactomes of EMT [7]. EMTome was examined to determine the gene signatures involved in EMT and subsequently aided in the analysis for RNA-Seq and meta-analysis.

BioPlex is a database that was used to predict protein–protein interactions of 14-3-3γ and the other EMT and autophagic proteins in cancer cells by utilizing datasets from collaboration between Harvard Medical School and Biogen [8]. Predicted proteins obtained from the BioPlex interactome were used to unravel the interactome of 14-3-3γ during EMT in cancer cells.

ANIA: Annotation and Integrated Analysis of the 14-3-3 interactome. ANIA was used to examine the possible human 14-3-3-binding phosphoproteins and phosphosites by utilizing datasets from studies that examined the phosphoproteins binding to 14-3-3 as well as high-throughput 14-3-3 affinity capture

together with mass spectrometry-based studies in identifying potential targets [9]. Proteins involved directly and indirectly in autophagy were used to examine their possible interactions with 14-3-3 by immunoprecipitation.

Survival Outcome Analytics using Clinical Databases. To assess the clinical relevance of *YWHAG* levels on patient survival, we interrogated four cancer cohort databases. First, by using The Cancer Genome Atlas Pan-Cancer Clinical Data Resource (TCGA-CDR) and Gene Expression database of Normal and Tumor tissues 2 (GENT2), *YWHAG* expression was examined in deposited tumor tissues as well as in cognate normal tissues. Next, we stratified the cancer patients into high and low *YWHAG*-expressing tumors. In the PrognoScan database [10], cohort studies that reported overall, distant metastasis-free, disease-specific and relapse-free survival and had significantly corrected *P* values were examined. The Prediction of Clinical Outcomes from Genomic Profiles (PRECOG) [11] evaluates the expression of 23,287 genes across 39 cancer types and clinical outcome data from 166 published expression datasets covering 18,000 tumors, thus extensively studying the associations between genomic profiles and cancer outcomes.

Human tumor biopsies. Human frozen tissue biopsies from the head and neck, breast, stomach and colon were purchased from Proteogenex (Proteogenex, California, USA) (**Table S6**).

Table S1. Patients with high *YWHAG*-expressing cancers (TCGA database) have a shorter median survival time

	Median Survival (months)	
	Low-*YWHAG*	High-*YWHAG*
BLCA	**47.43** (184)	**31.63** (220)
BRCA	**142.23** (283)	**124.53** (806)
CECS	**40.33** (227)	**17.43** (77)
COAD	# (115)	**92.14** (222)
HNSC	**58.73** (363)	**31.37** (136)
KIRC	**118.47** (393)	**70.17** (137)
KIRP	**89.47** (125)	**48.93** (162)
LGG	**145.05** (168)	**73.48** (336)
LIHC	**70.53** (128)	**52** (242)
LUAD	**55.1** (336)	**32.9** (168)
PRAD	# (245)	**113.98** (243)
SARC	**82.13** (68)	**65.1** (191)
SKMC	**103.26**(229)	**65.88** (229)
STAD	**43.13** (277)	**22.5** (94)
UCEC	**108.37** (236)	**37.57** (306)
LUSC	54.67 (333)	55.2 (162)
OV	45.17(123)	43.97(250)

Median survival cannot be determined because it did not reach 50% survival probability.
The number of patients is indicated in parentheses.
Tumor types with <200 cancer patients were underpowered and not included in the analysis.

Table S2. Ten cohorts from the PrognoScan database showed that high *YWHAG* expression was associated with poor prognosis.

DATASET	CANCER TYPE	ENDPOINT	COHORT	N	CORRECTED P-VALUE	ln(HR-high/HR-low)	COX P-VALUE	ln(HR)	HR [95% CI-low CI-upp]
GSE31210	Lung cancer	Relapse Free Survival	NCCRI	204	1.71E-05	1.314	7.26E-07	4.061	58.02 [11.64 - 289.26]
		Overall Survival	NCCRI	204	1.12E-03	1.522	5.94E-04	3.699	40.40 [4.89 - 333.62]
GSE16581	Brain cancer	Overall Survival	UCLA	67	8.80E-05	2.538	1.88E-02	3.854	47.17 [1.89 - 1176.18]
GSE12276	Breast cancer	Relapse Free Survival	EMC	204	2.46E-04	0.650	1.18E-03	1.041	2.83 [1.51 - 5.31]
GSE1456-GPL97	Breast cancer	Relapse Free Survival	Stockholm (1994-1996)	159	3.34E-03	1.182	1.09E-03	1.463	4.32 [1.80 - 10.40]
		Disease Specific Survival	Stockholm (1994-1996)	159	9.45E-03	1.391	3.91E-03	1.544	4.68 [1.64 - 13.37]
		Overall Survival	Stockholm (1994-1996)	159	0.048	1.110	0.057	0.868	2.38 [0.97 - 5.83]
GSE9893	Breast cancer	Overall Survival	Montpellier, Bordeaux, Turin (1989-2001)	155	0.012	0.998	0.011	0.228	1.26 [1.05 - 1.50]
GSE9195	Breast cancer	Distant Metastasis Free Survival	GUYT2	77	0.017	2.221	0.047	1.367	3.92 [1.02 - 15.14]
		Relapse Free Survival	GUYT2	77	0.036	2.060	0.084	1.101	3.01 [0.86 - 10.47]
GSE19234	Skin cancer	Overall Survival	NYU	38	0.020	1.636	0.012	2.111	8.26 [1.59 - 42.85]
GSE6532-GPL570	Breast cancer	Distant Metastasis Free Survival, Relapse Free Survival	GUYT	87	0.027	1.232	0.267	0.685	1.98 [0.59 - 6.65]
GSE4922-GPL97	Breast cancer	Disease Free Survival	Uppsala (1987-1989)	249	0.029	0.694	0.036	0.825	2.28 [1.05 - 4.94]
GSE17536	Colorectal cancer	Overall Survival	MCC	177	0.041	2.471	0.076	0.875	2.40 [0.91 - 6.30]

Ten cohort studies covering various types of cancers and end points from the PrognoScan database illustrated the hazard ratio of cancer-specific events. Observations demonstrated that high *YWHAG* expression is correlated with poorer prognosis in patients. HR, hazard ratio; CI, confidence interval.

Table S3. Global unweighted meta-Z score of all cancers from the PRECOG database

Gene	Z score
YWHAZ	4.16
YWHAG	4.13
YWHAQ	2.68
YWHAS	2.38
YWHAB	2.31
YWHAE	1.71
YWHAH	0.76
AKT1	2.55
SRC	1.95
MDM2	1.79
MYC	1.76
EGFR	1.71

The PRECOG database encompasses 166 cancer expression datasets, including overall survival data for ~18,000 patients diagnosed with 39 distinct malignancies.

Table S4. Antibodies used for immunoblotting, immunoprecipitation and immunofluorescence.

Antibodies	Species	Vendor	Catalog Number
14-3-3ε	Rabbit	Cell Signaling Technology	9635S
14-3-3η	Rabbit	Cell Signaling Technology	5521S
14-3-3α/β	Rabbit	Cell Signaling Technology	9636S
14-3-3ζ/δ	Rabbit	Cell Signaling Technology	7413S
14-3-3τ	Rabbit	Cell Signaling Technology	9638S
E-Cadherin ®	Rabbit	Cell Signaling Technology	3195S
E-Cadherin	Mouse	Cell Signaling Technology	14472
Vimentin ®	Rabbit	Cell Signaling Technology	5741S
N-Cadherin	Mouse	Cell Signaling Technology	14215S
NOX4	Rabbit	Cell Signaling Technology	14215S
Snai1	Rabbit	Cell Signaling Technology	3879S
Twist	Rabbit	Cell Signaling Technology	69366S
14-3-3γ	Rabbit	Abcam	Ab237732
GPX1	Rabbit	Abcam	Ab22604
NRF2	Rabbit	Abclonal	A2201
ATG5	Rabbit	Novus Biologicals	NB110-53818
Beclin	Rabbit	Invitrogen	MA5-15825
LC3A/B	Rabbit	MBL	M152-3
ULK1 (D8H5)	Rabbit	Cell Signaling Technology	8054S
FIP200	Rabbit	Cell Signaling Technology	12436S
Tubulin	Mouse	DHSB	E7
IgG	Rabbit	Proteintech	30000-0-AP
Alexa Fluor™ 488	Goat	Thermo Scientific	A21042
Alex Fluor™ 594	Goat	Thermo Scientific	A32740
IRDye® 680RD AntiMouse IgG (H+L)	Goat	LI-COR Biosciences	LIC-92568070
IRDye® 680RD AntiRabbit IgG (H+L)	Goat	LI-COR Biosciences	LIC-92568071
IRDye® 800RD AntiMouse IgG (H+L)	Goat	LI-COR Biosciences	LIC-92632210
IRDye® 800RD AntiRabbit IgG (H+L)	Goat	LI-COR Biosciences	LIC92532211

Table S5. List of qPCR Primers.

Primers (Human)	Accession number	Forward Primer Sequence (5' to 3')	Reverse Primer Sequence (5' to 3')	Amplicon length (bp)
18S	NR_146119.1	GTAACCCGTTGAACCCCATT	CCATCCAATCGGTAGTAGCG	151
ATG5	NM_004849.4	TCTGCACTGTCCATCTAAGGATG	TCCGATTGATGGCCCAAAACTGG	176
BECN1	NM_003766.5	CTGGACACGAGTTTCAAGATCCT	TGTGGTAAGTAATGGAGCTGTGAGTT	69
CDH1	NM_004360.3	CCCACCACGTACAAGGGTC	CTGGGGTATTGGGGGCATC	94
CDH2	NM_001792.5	TGTGCATGAAGGACAGCCTC	CTGCCACTTGCCACTTTTCC	212
GPX1	NM_201397.3	TCTGTTGCTCGTAGCTGCTG	CTATGAGTCACCGGGATTTTG	77
GPX2	NM_002083.4	GAGCTGCAATGCCGCTTTCC	TGGACAAGGGTGAAGGTGGGC	155
MAP1LC3B	NM_022818.5	AGCAGCATCCAACCAAAATC	CTGTGTCCGTTCACCAACAG	187
NOX1	NM_007052.5	CTGCTTCCTGTGTGTCGCAA	AGGCAGATCATATAGGCCACC	128
NOX2	NM_000397.4	ACATTCAACCTCTGCCACCA	CCCCAGCCAAACCAGAAT	79
NOX4	NM_016931.5	GGGCCGGCGGCATGGCTGT	CGGCACATGGGTAAAAGGAT	241
NQO1	NM_000903.3	GAAGAGCACTGATCGTACTGGC	GGATACTGAAAGTTCGCAGGG	196
NRF2	NM_006164.5	ACACGGTCCACAGCTCATC	TGCCTCCAAAGTATGTCAATCA	96
SNAI1	NM_005985.4	GCCTTCAACTGCAAATACTGC	CTTCTTGACATCTGAGTGGGTC	249
SQSTM1	NM_003900.5	AGGCGCACTACCGCGAT	CGTCACTGGAAAAGGCAACC	51
TBP	NM_001172085	GTGGGGAGCTGTGATGTGAA	GGGAGGCAAGGGTACATGAG	271
VIM	NM_003380.5	TGGTCTAACGGTTTCCCCTA	GACCTCGGAGCGAGAGTG	86
YWHAB	NM_003404.5	GGCAAAGAGTACCGTGAGAAG	CTGGTTGTGTAGCATTGGGAATA	103
YWHAE	NM_006761.5	GATTCGGGAATATCGGCAAATGG	GCTGGAATGAGGTGTTTGTCC	90
YWHAG	NM_012479.4	AGCCACTGTCGAATGAGGAAC	CTGCTCAATGCTACTTGATGACC	101
YWHAH	NM_003405.4	TGGCTGATGGAAACGAAAAGAA	CCTCTGCTAAGTAGGCGGTAGT	199
YWHAQ	NM_006826.4	AGGGTCATCTCTAGCATCGAG	CCACTTTCTCCCGATTAGTCCTT	83
YWHAZ	NM_003406.4	CCTGCATGAAGTCTGTAACTGAG	GACCTACGGGCTCCCTACAACA	101
ZEB1	NM_001128128.3	GCCAACAGACCAGACAGTGTT	TCTTGCCCTTCCTTTCCTGTG	96
**Primers (Mouse)**	**Accession number**	**Forward Primer Sequence (5' to 3')**	**Reverse Primer Sequence (5' to 3')**	**Amplicon length (bp)**
18s	NR_003278.3	GTAACCCGTTGAACCCCATT	CCATCCAATCGGTAGTAGGG	151
Atg5	NM_053069.6	TGTGCTTCGAGATGTGTGGTT	GTCAAATAGCTGACTCTTGGCAA	120
Becn1	NM_019584.4	ATGGAGGGGTCTAAGGCGTC	TCCTCTCCTGAGTTAGCCTCT	197

Map1lc3b	NM_026160.5	TTATAGAGCGATACAAGGGGGAG	CGCCGTCTGATTATCTTGATGAG	109
Sqstm1	NM_011018.3	AGGATGGGGACTTGGTTGC	TCACAGATCACATTGGGGTGC	178
Ywhag	NM_018871.3	GTGACCGAGCTGAACGAAC	GATGCTGCTGATGACCCTCC	111

Table S6: Clinical information of human tumor biopsies.

Sample source	Sex	Age	Diagnosis	Histological diagnosis	Grade	TNM	Stage
Stomach	M	64	Primary	Adenocarcinoma	G2	T2N0M0	IB
Stomach	M	58	Primary	Adenocarcinoma	G3	T3N2M0	IIIA
Colon	M	66	Primary	Adenocarcinoma	G1	T2N0M0	I
Colon	M	80	Primary	Adenocarcinoma	G1	T3N1M0	IIIB
Colon to lung	F	42	Metastases	Adenocarcinoma	N/A	Recurrent	M
Breast	F	51	Primary	Infiltrating ductal carcinoma	G2	T1cN0M0	I
Breast to ovary	F	59	Metastases	Infiltrating ductal carcinoma	N/A	Recurrent	M
Head & Neck	M	58	Primary	Squamous cell carcinoma	G1	T2N0M0	I
Head & Neck	M	64	Primary	Squamous cell carcinoma	G2	T2N1M0	III

References

[1] Y. Liao, G. K. Smyth, W. Shi, *Bioinformatics* **2014**, *30* (7), 923, https://doi.org/10.1093/bioinformatics/btt656.

[2] A. Brionne, A. Juanchich, C. Hennequet-Antier, *BioData Min* **2019**, *12*, 16, https://doi.org/10.1186/s13040-019-0204-1.

[3] J. Reimand, R. Isserlin, V. Voisin, M. Kucera, C. Tannus-Lopes, A. Rostamianfar, L. Wadi, M. Meyer, J. Wong, C. Xu, D. Merico, G. D. Bader, *Nat Protoc* **2019**, *14* (2), 482, https://doi.org/10.1038/s41596-018-0103-9.

[4] A. Kramer, J. Green, J. Pollard, Jr., S. Tugendreich, *Bioinformatics* **2014**, *30* (4), 523, https://doi.org/10.1093/bioinformatics/btt703.

[5] a) G. Bindea, B. Mlecnik, H. Hackl, P. Charoentong, M. Tosolini, A. Kirilovsky, W. H. Fridman, F. Pages, Z. Trajanoski, J. Galon, *Bioinformatics* **2009**, *25* (8), 1091, https://doi.org/10.1093/bioinformatics/btp101; b) P. Shannon, A. Markiel, O. Ozier, N. S. Baliga, J. T. Wang, D. Ramage, N. Amin, B. Schwikowski, T. Ideker, *Genome Res* **2003**, *13* (11), 2498, https://doi.org/10.1101/gr.1239303.

[6] C. H. Chin, S. H. Chen, H. H. Wu, C. W. Ho, M. T. Ko, C. Y. Lin, *BMC Syst Biol* **2014**, *8 Suppl 4* (Suppl 4), S11, https://doi.org/10.1186/1752-0509-8-S4-S11.

[7] S. V. Vasaikar, A. P. Deshmukh, P. den Hollander, S. Addanki, N. A. Kuburich, S. Kudaravalli, R. Joseph, J. T. Chang, R. Soundararajan, S. A. Mani, *Br J Cancer* **2021**, *124* (1), 259, https://doi.org/10.1038/s41416-020-01178-9.

[8] E. L. Huttlin, L. Ting, R. J. Bruckner, F. Gebreab, M. P. Gygi, J. Szpyt, S. Tam, G. Zarraga, G. Colby, K. Baltier, R. Dong, V. Guarani, L. P. Vaites, A. Ordureau, R. Rad, B. K. Erickson, M. Wuhr, J. Chick, B. Zhai, D. Kolippakkam, J. Mintseris, R. A. Obar, T. Harris, S. Artavanis-Tsakonas, M. E. Sowa, P. De Camilli, J. A. Paulo, J. W. Harper, S. P. Gygi, *Cell* **2015**, *162* (2), 425, https://doi.org/10.1016/j.cell.2015.06.043.

[9] M. Tinti, F. Madeira, G. Murugesan, G. Hoxhaj, R. Toth, C. Mackintosh, *Database (Oxford)* **2014**, *2014*, bat085, https://doi.org/10.1093/database/bat085.

[10] H. Mizuno, K. Kitada, K. Nakai, A. Sarai, *BMC Med Genomics* **2009**, *2*, 18, https://doi.org/10.1186/1755-8794-2-18.

[11] A. J. Gentles, A. M. Newman, C. L. Liu, S. V. Bratman, W. Feng, D. Kim, V. S. Nair, Y. Xu, A. Khuong, C. D. Hoang, M. Diehn, R. B. West, S. K. Plevritis, A. A. Alizadeh, *Nat Med* **2015**, *21* (8), 938, https://doi.org/10.1038/nm.3909.

Supplementary Figure Legends

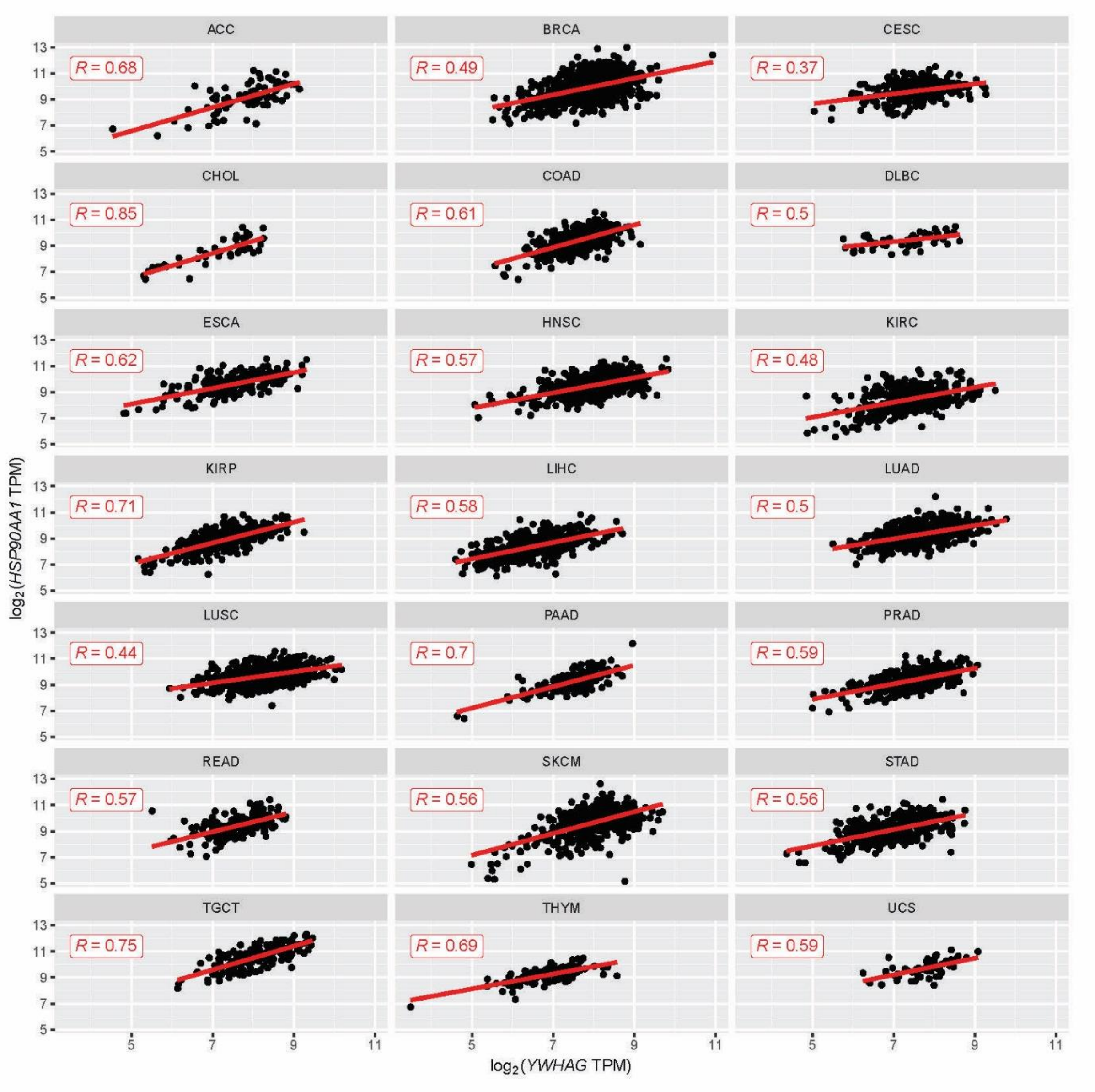

Figure S1. Correlation of *YWHAG* and *HSP90AA1* expression in 21 TCGA cancer types.

Log_2 (normalized count [transcript per million; TPM]) of *HSP90AA1* and *YWHAG* were retrieved from 21 TCGA tumor types, including adrenocortical carcinoma (ACC), breast invasive carcinoma (BRCA), cervical squamous cell carcinoma and endocervical adenocarcinoma (CECS), cholangio carcinoma (CHOL), lymphoid neoplasm diffuse large B-cell lymphoma (DLBC), esophageal carcinoma (ESCA), head and neck squamous cell carcinoma (HNSC), kidney renal clear cell carcinoma (KIRC), kidney renal papillary cell carcinoma (KIRP), liver hepatocellular carcinoma (LIHC), lung adenocarcinoma (LUAD), lung squamous cell carcinoma (LUSC), pancreatic adenocarcinoma (PAAD), prostate adenocarcinoma (PRAD), rectum adenocarcinoma (READ), skin cutaneous melanoma (SKCM), stomach adenocarcinoma (STAD), testicular germ cell tumors (TGCT), thymoma (THYM), and uterine carcinosarcoma (UCS).

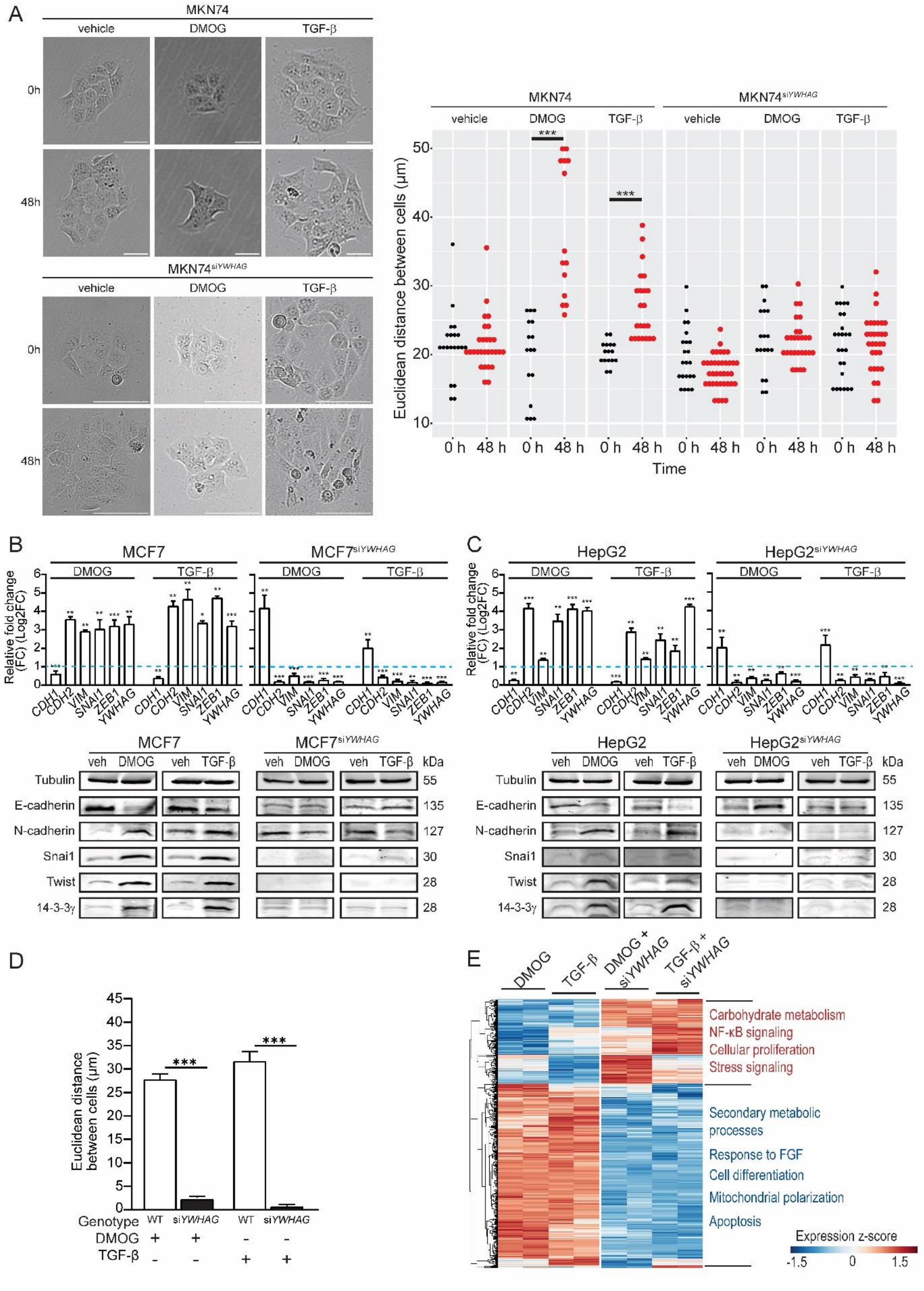
A
MKN74
vehicle
DMOG
TGF-β
0h
48h
MKN74siYWHAG
Euclidean distance between cells (μm)
Time
B
MCF7
MCF7siYWHAG
Relative fold change (FC) (Log2FC)
CDH1
CDH2
VIM
SNAI1
ZEB1
YWHAG
veh
kDa
Tubulin
E-cadherin
N-cadherin
Snai1
Twist
14-3-3γ
C
HepG2
HepG2siYWHAG
D
Euclidean distance between cells (μm)
Genotype
WT
siYWHAG
E
DMOG + siYWHAG
TGF-β + siYWHAG
Carbohydrate metabolism
NF-κB signaling
Cellular proliferation
Stress signaling
Secondary metabolic processes
Response to FGF
Cell differentiation
Mitochondrial polarization
Apoptosis
Expression z-score

Figure S2. *YWHAG* deficiency delays DMOG- and TGF-β-induced EMT in cancer cells.

(A, D) Representative brightfield images (A) and Euclidean distance (A, D) of untreated, DMOG (0.5 mM)- and TGF-β1 (10 ng/mL)-treated MKN74 and MKN74$^{si\textit{YWHAG}}$ cells. Scale bar = 100 μM.

(B-C) qPCR (top) and immunoblots (bottom) of epithelial and mesenchymal markers in MCF7 and MCF7$^{si\textit{YWHAG}}$ (B) and HepG2 and HepG2$^{si\textit{YWHAG}}$ (C) cells during EMT induced by DMOG or TGF-β. The blue dotted line represents the relative expression of untreated cells.

(E) Heatmaps and Gene Ontology analysis of DEGs revealed functional changes in MKN74 and MKN74$^{si\textit{YWHAG}}$ cells during EMT induced by DMOG or TGF-β1.

Data, wherever applicable, are represented as the mean ± SD from at least three independent experiments. ***p < 0.001, **p < 0.01, *p < 0.05 and n.s. denotes not significant (Mann–Whitney U test).

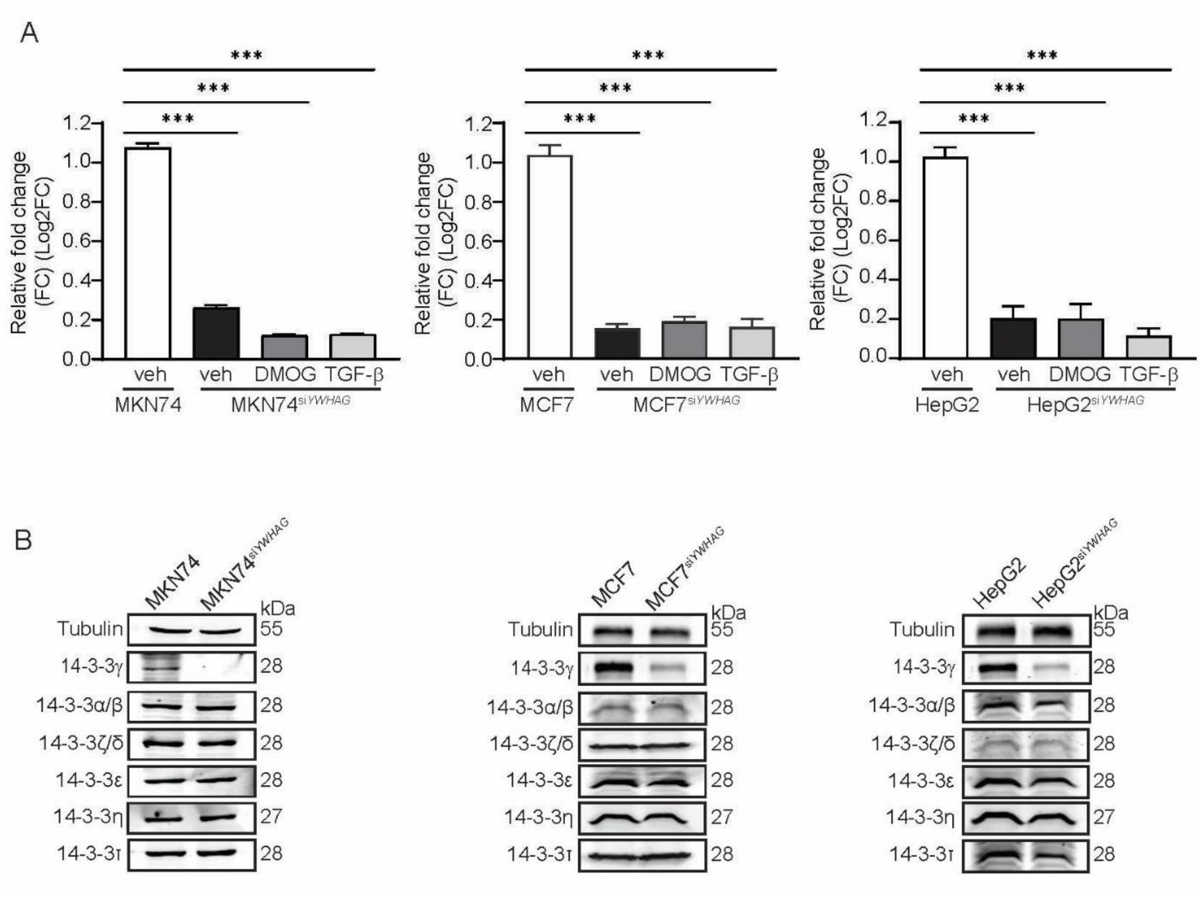

Figure S3. *YWHAG* knockdown efficiency and specificity in cancer cells.

(A) Relative fold change in *YWHAG* mRNA levels in wild-type MKN74, MCF7 and HepG2 cells, as well as untreated, DMOG- or TGF-β-treated *YWHAG*-knockdown cells (MKN74$^{\mathrm{si}\textit{YWHAG}}$, MCF7$^{\mathrm{si}\textit{YWHAG}}$ and HepG2$^{\mathrm{si}\textit{YWHAG}}$) at 48 h after siRNA transfection. TBP and 18S were used as housekeeping genes.

(B) Representative immunoblots of 14-3-3 family members in wild-type and *YWHAG*-knockdown cancer cells. The β-tubulin protein was used as a loading and transfer control from the same samples.

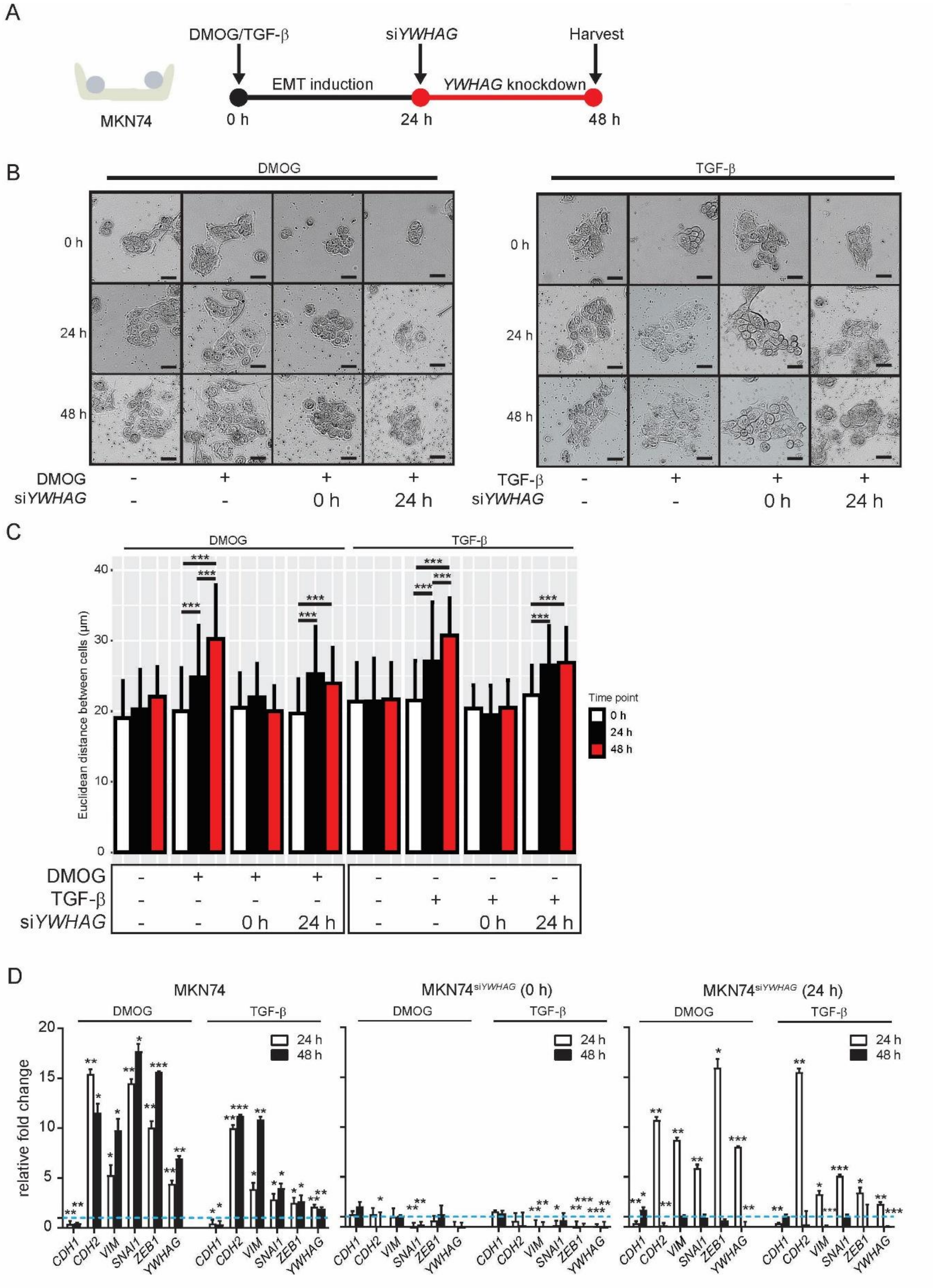

A
DMOG/TGF-β
siYWHAG
Harvest
EMT induction
YWHAG knockdown
MKN74
0 h
24 h
48 h
B
DMOG
TGF-β
0 h
24 h
48 h
DMOG - + + +
siYWHAG - - 0 h 24 h
TGF-β - + + +
siYWHAG - - 0 h 24 h
C
DMOG
TGF-β
Euclidean distance between cells (μm)
Time point
0 h
24 h
48 h
DMOG - + + + - - - -
TGF-β - - - - - + + +
siYWHAG - - 0 h 24 h - - 0 h 24 h
D
MKN74
MKN74siYWHAG (0 h)
MKN74siYWHAG (24 h)
DMOG
TGF-β
relative fold change
24 h
48 h
CDH1
CDH2
VIM
SNAI1
ZEB1
YWHAG

Figure S4. *YWHAG* knockdown ceases active EMT in MKN74.

(A) Schematic diagram of the experimental setup to study the effect of *YWHAG* knockdown in MKN74 undergoing active EMT. EMT was induced using DMOG (0.5 mM) or TGF-β1 (10 ng/mL) for 24 h. Successful EMT was confirmed by the upregulation of mesenchymal-associated genes and downregulation of epithelial genes. After 24 h of EMT induction, the cells were transfected with 10 nM si*YWHAG* and maintained for another 24 h to examine the effect of *YWHAG* knockdown on mesenchymal and epithelial gene expression. Three control groups were carried out concurrently, including untreated cells, EMT cells, and MKN74$^{si\textit{YWHAG}}$ at 0 h.

(B-C) Representative brightfield images (C) and Euclidean distance (D) of untreated, DMOG- and TGF-β1-treated MKN74 and MKN74$^{si\textit{YWHAG}}$ transfected at 0 h or 24 h. Scale bar = 100 μM.

(D) Gene expression of epithelial and mesenchymal markers in DMOG- or TGF-β-induced EMT in MKN74 (left), MKN74$^{si\textit{YWHAG}}$ transfected at 0 h (mid) and MKN74$^{si\textit{YWHAG}}$ transfected at 24 h (right). The blue dotted line represents the relative expression of untreated cells. *TBP* and *18S* were used as housekeeping genes for qPCR.

Data, wherever applicable, are represented as the mean ± SD from at least three independent experiments. ***p < 0.001, **p < 0.01, *p < 0.05 and n.s. denotes not significant (Mann–Whitney U test).

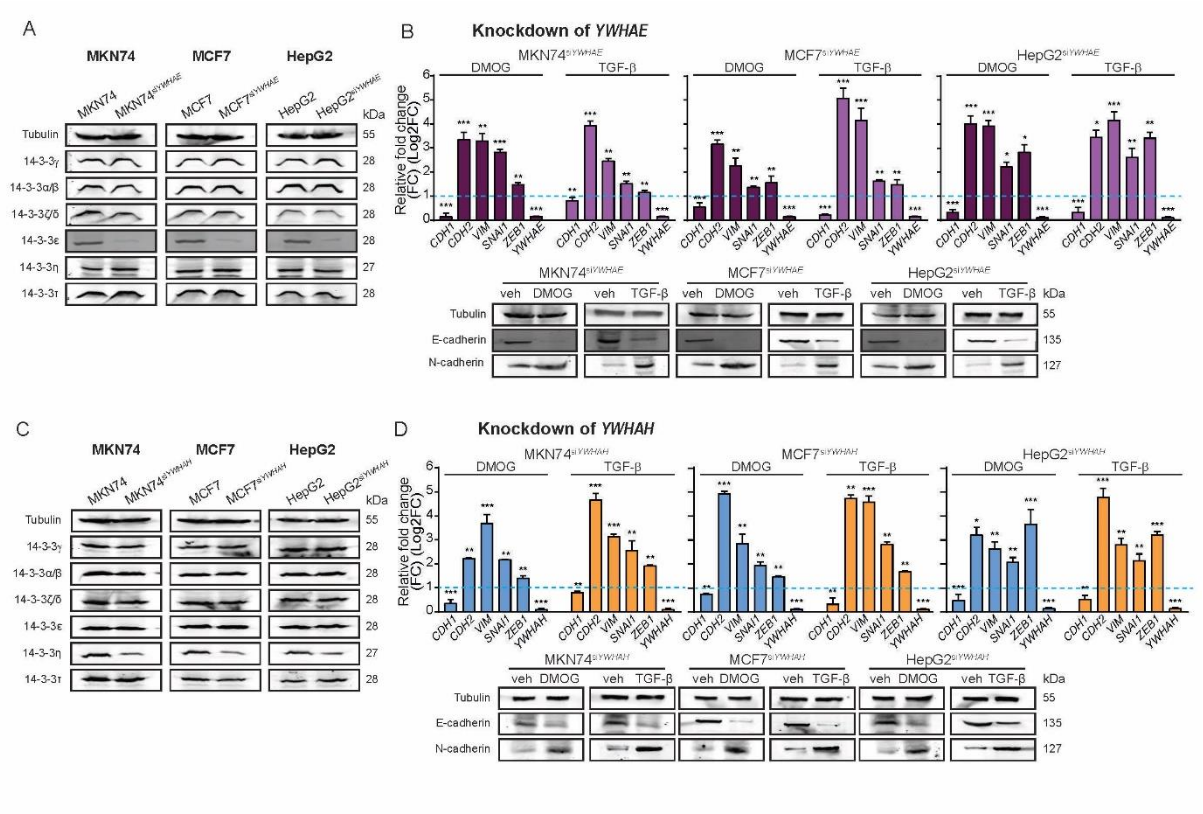

Figure S5. Effects of *YWHAE* and *YWHAH* knockdown on EMT.

(A, C) Representative immunoblots of seven 14-3-3 isoforms in *YWHAE*-knockdown (denoted as MKN74$^{\text{si}\mathit{YWHAE}}$, MCF7$^{\text{si}\mathit{YWHAE}}$ and HepG2$^{\text{si}\mathit{YWHAE}}$) (A) and *YWHAH*-knockdown (denoted MKN74$^{\text{si}\mathit{YWHAH}}$, MCF7$^{\text{si}\mathit{YWHAH}}$ and HepG2$^{\text{si}\mathit{YWHAH}}$) (C) MKN74, MCF7 and HepG2 cells. The β-tubulin protein served as a loading and transfer control for immunoblot analysis from the same samples.

(B, D) Relative mRNA (top) and protein (bottom) levels of EMT markers in DMOG- and TGF-β-treated MKN74$^{\text{si}\mathit{YWHAE}}$, MCF7$^{\text{si}\mathit{YWHAE}}$ and HepG2$^{\text{si}\mathit{YWHAE}}$ cells (B) and MKN74$^{\text{si}\mathit{YWHAH}}$, MCF7$^{\text{si}\mathit{YWHAH}}$ and HepG2$^{\text{si}\mathit{YWHAH}}$ cells (D).

Data, wherever applicable, are represented as the mean ± SD from at least three independent experiments. ***$p < 0.001$, **$p < 0.01$, *$p < 0.05$ and n.s. denotes not significant (Mann–Whitney U test).

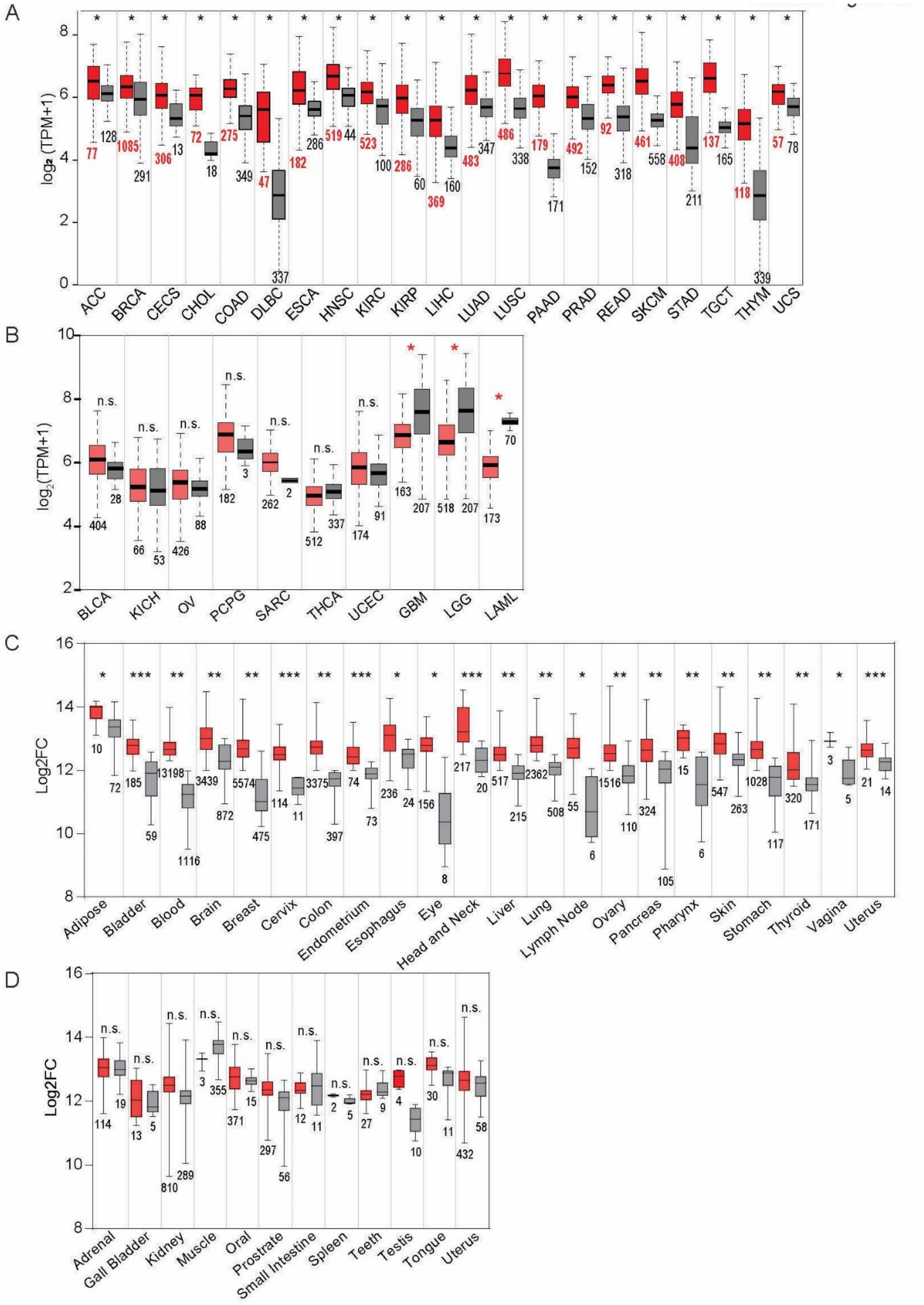
A
log2 (TPM+1)
ACC BRCA CECS CHOL COAD DLBC ESCA HNSC KIRC KIRP LIHC LUAD LUSC PAAD PRAD READ SKCM STAD TGCT THYM UCS
B
log2(TPM+1)
BLCA KICH OV PCPG SARC THCA UCEC GBM LGG LAML
C
Log2FC
Adipose Bladder Blood Brain Breast Cervix Colon Endometrium Esophagus Eye Head and Neck Liver Lung Lymph Node Ovary Pancreas Pharynx Skin Stomach Thyroid Vagina Uterus
D
Log2FC
Adrenal Gall Bladder Kidney Muscle Oral Prostrate Small Intestine Spleen Teeth Testis Tongue Uterus

Figure S6. High *YWHAG* is a common feature in many tumor types.

(A-D) Tumor type (red) which showed statistically significant higher *YWHAG* mRNA level (A, C) or which did not show significant differences (B, D) compared with cognate normal tissues (in gray) obtained from TCGA (A, B) and GENT2 (C, D) databases. TPM denotes transcripts per million. Values below the box-whisker plots represent the number of patients from whom the tumour samples were derived. * p<0.01. n.s. denotes no significance.

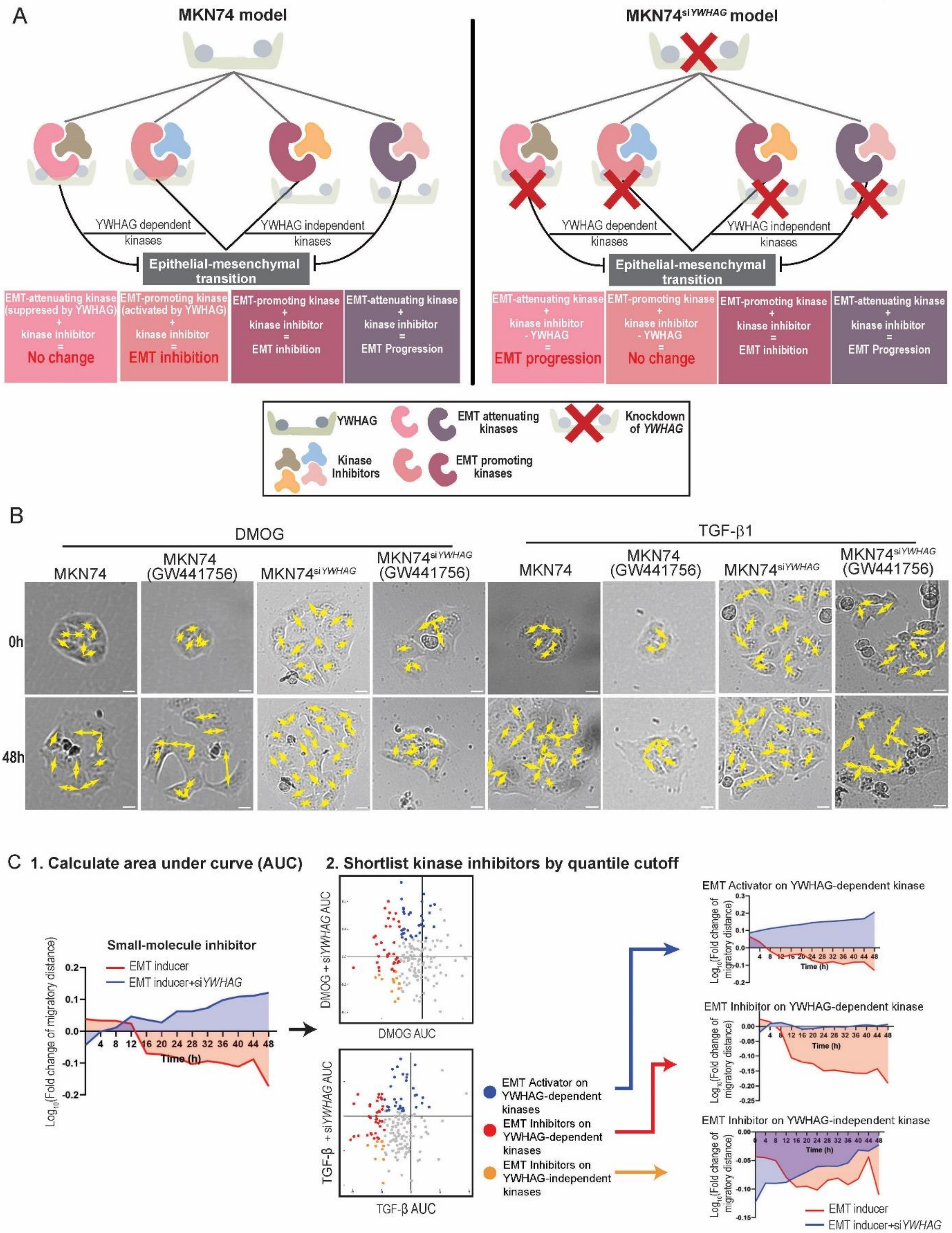
A
MKN74 model
MKN74siYWHAG model
YWHAG dependent kinases
YWHAG independent kinases
Epithelial-mesenchymal transition
EMT-attenuating kinase (suppresed by YWHAG) + kinase inhibitor = No change
EMT-promoting kinase (activated by YWHAG) + kinase inhibitor = EMT inhibition
EMT-promoting kinase + kinase inhibitor = EMT inhibition
EMT-attenuating kinase + kinase inhibitor = EMT Progression
EMT-attenuating kinase + kinase inhibitor - YWHAG = EMT progression
EMT-promoting kinase + kinase inhibitor - YWHAG = No change
YWHAG
EMT attenuating kinases
Knockdown of YWHAG
Kinase Inhibitors
EMT promoting kinases
B
DMOG
TGF-β1
MKN74
MKN74 (GW441756)
MKN74siYWHAG
MKN74siYWHAG (GW441756)
0h
48h
C 1. Calculate area under curve (AUC)
2. Shortlist kinase inhibitors by quantile cutoff
Small-molecule inhibitor
EMT inducer
EMT inducer+siYWHAG
Log10(Fold change of migratory distance)
Time (h)
DMOG + siYWHAG AUC
DMOG AUC
TGF-β + siYWHAG AUC
TGF-β AUC
EMT Activator on YWHAG-dependent kinases
EMT Inhibitors on YWHAG-dependent kinases
EMT Inhibitors on YWHAG-independent kinases
EMT Activator on YWHAG-dependent kinase
EMT Inhibitor on YWHAG-dependent kinase
EMT Inhibitor on YWHAG-independent kinase
EMT inducer
EMT inducer+siYWHAG

Figure S7. Interpretation of the kinase inhibitor screen during EMT of MKN74 cells

(A) Schematic diagram of the kinase inhibitor screens in MKN74 and MKN74$^{si\textit{YWHAG}}$ cells during EMT induction by DMOG or TGF-β. Illustration included the possible correlation between *YWHAG* dependency and the outcomes on migratory distance (EMT progression, EMT inhibition or no change).

(B) Illustration of Euclidean distances between multivariate centroids of the cell populations at 0 h and 48 h in MKN74 and MKN74$^{si\textit{YWHAG}}$ cells treated with EMT inducters and kinase inhibitors.

(C) Schematic diagram of the calculation and classification of kinase inhibitors in the screens. For every kinase inhibitor, the average Euclidean distance, which was measured every 4 h for 48 h after EMT induction, was considered the migratory distance. The log fold change in migratory distance between treated (with kinase inhibitor) and DMSO-treated MKN74 cells was calculated, and the temporal changes were plotted. The AUC, which reflects the overall effect of kinase inhibitors on EMT, was determined and compared between wild-type MKN74 and MKN74$^{si\textit{YWHAG}}$ cells. Kinase inhibitors were classified into EMT activators on *YWHAG*-dependent kinases or EMT inhibitors on *YWHAG*-dependent/independent kinases based on the similarity/difference in AUC between genotypes. Examples of temporal changes in the log fold change of migratory distance for each classification are shown.

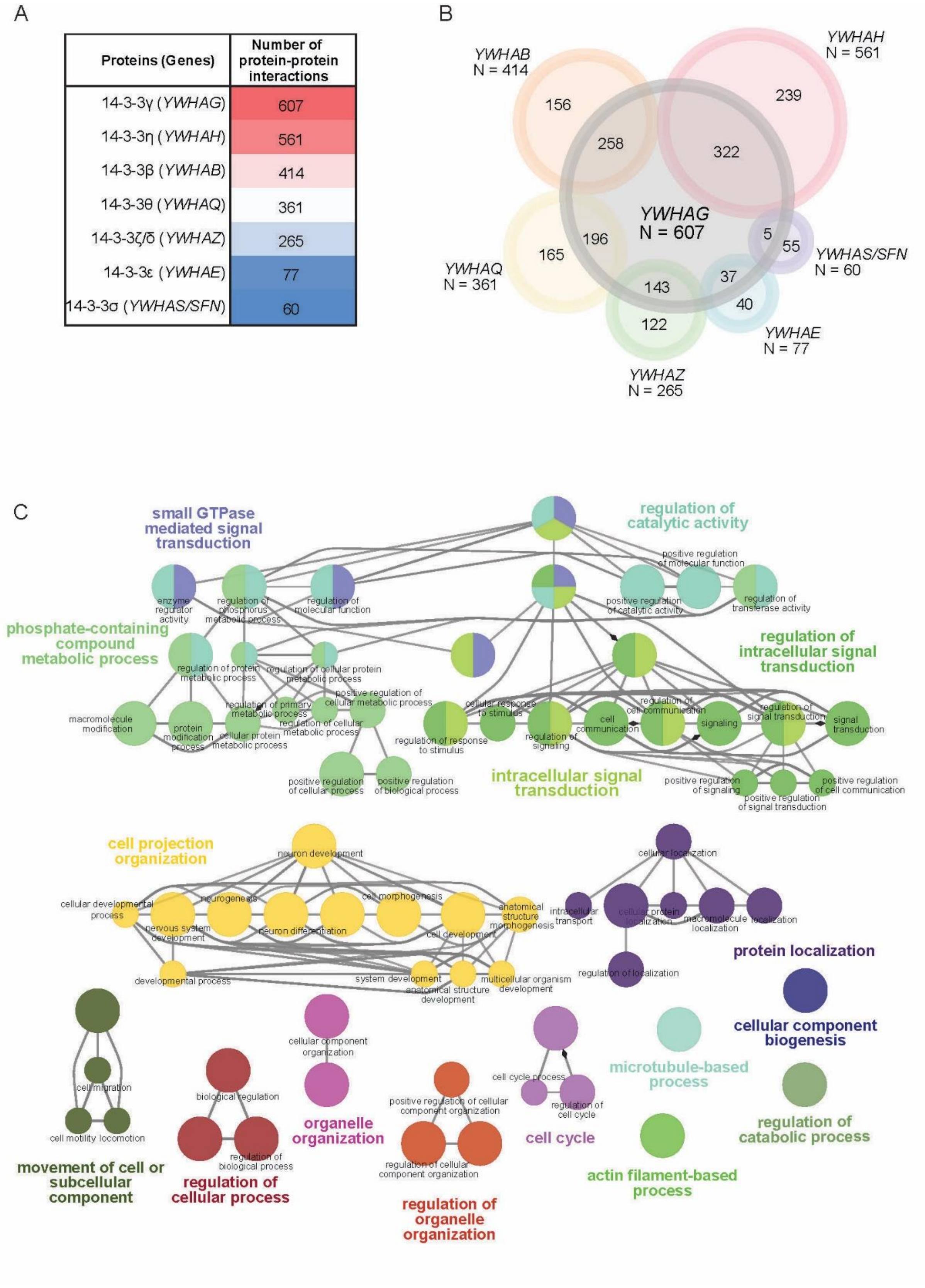

A

Proteins (Genes)	Number of protein-protein interactions
14-3-3γ (*YWHAG*)	607
14-3-3η (*YWHAH*)	561
14-3-3β (*YWHAB*)	414
14-3-3θ (*YWHAQ*)	361
14-3-3ζ/δ (*YWHAZ*)	265
14-3-3ε (*YWHAE*)	77
14-3-3σ (*YWHAS/SFN*)	60

Figure S8. Protein–protein interactomes of 14-3-3 family members.

(A) Table shows the number of interacting proteins with the different isoforms of 14-3-3 isoforms derived from the BioPlex Interactome database.

(B) Venn diagrams showing common interacting proteins among the different 14-3-3 isoforms. The number of interactomes for each 14-3-3 isoform is indicated in parentheses.

(C) Gene ontologies of the interactomes of 14-3-3γ. The size and color in circle correspond to relative number of interacting proteins in the gene ontologies of the same color.

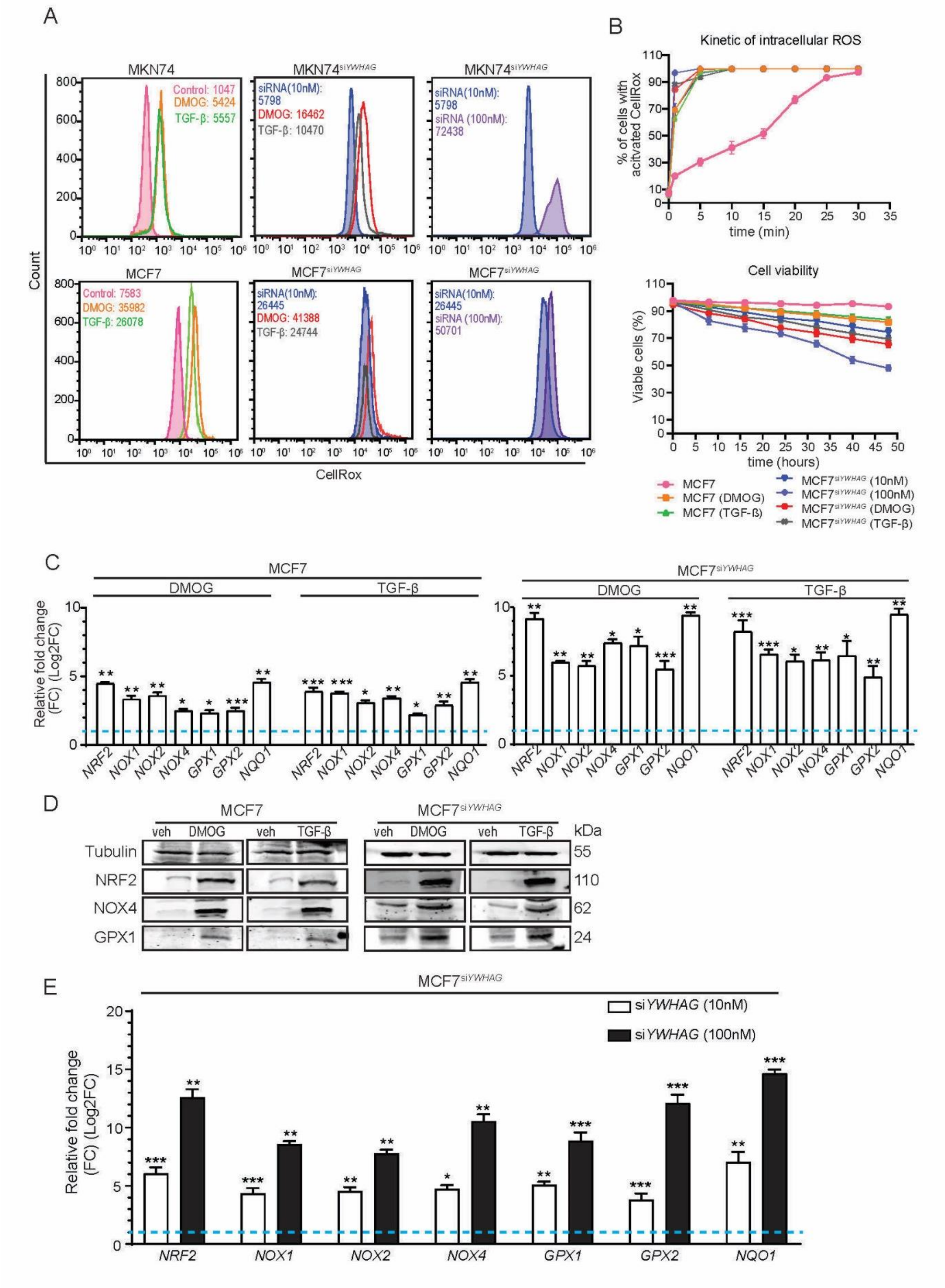
A
MKN74
Control: 1047
DMOG: 5424
TGF-β: 5557
MKN74siYWHAG
siRNA(10nM): 5798
DMOG: 16462
TGF-β: 10470
MKN74siYWHAG
siRNA(10nM): 5798
siRNA (100nM): 72438
MCF7
Control: 7583
DMOG: 35982
TGF-β: 26078
MCF7siYWHAG
siRNA(10nM): 26445
DMOG: 41388
TGF-β: 24744
MCF7siYWHAG
siRNA(10nM): 26445
siRNA (100nM): 50701
Count
CellRox
B
Kinetic of intracellular ROS
% of cells with acitvated CellRox
time (min)
Cell viability
Viable cells (%)
time (hours)
MCF7
MCF7 (DMOG)
MCF7 (TGF-ß)
MCF7siYWHAG (10nM)
MCF7siYWHAG (100nM)
MCF7siYWHAG (DMOG)
MCF7siYWHAG (TGF-β)
C
MCF7
DMOG
TGF-β
MCF7siYWHAG
Relative fold change (FC) (Log2FC)
NRF2
NOX1
NOX2
NOX4
GPX1
GPX2
NQO1
D
MCF7
MCF7siYWHAG
veh
DMOG
TGF-β
kDa
Tubulin
NRF2
NOX4
GPX1
55
110
62
24
E
MCF7siYWHAG
siYWHAG (10nM)
siYWHAG (100nM)
Relative fold change (FC) (Log2FC)
NRF2
NOX1
NOX2
NOX4
GPX1
GPX2
NQO1

Figure S9. *YWHAG* deficiency induces oxidative stress in MCF7 cells.

(A) Intracellular ROS profiles of MKN74 and MCF7 transfected with different concentrations of siYWHAG (0, 10 nM and 100 nM) with or without the co-treatment of EMT inducers, DMOG- and TGF-β.

(B) Graph showing the kinetics of intracellular ROS (left) and cell viability (right) in untreated, DMOG- and TGF-β-treated MCF7, MCF7$^{\mathrm{si}\textit{YWHAG}}$, MCF7$^{\mathrm{si}\textit{YWHAG}}$ (10nM) and MCF7$^{\mathrm{si}\textit{YWHAG}}$ (100nM) cells over a period of 48 h at 8-h intervals.

(C-D) Relative mRNA and protein expression of oxidative stress markers in MCF7 cells treated with DMOG and TGF-β. TBP and 18S were used as housekeeping genes for qPCR. The blue dotted line represents the relative expression of untreated cells.

(E) Relative mRNA expression of oxidative stress markers in MCF7$^{\mathrm{si}\textit{YWHAG}}$ (10nM) and MCF7$^{\mathrm{si}\textit{YWHAG}}$ (100nM) cells.

In (C) and (E), the blue dotted lines represent the relative expression of untreated cells. Data are represented as the mean±SD from at least three independent experiments. ***$p < 0.001$, **$p < 0.01$, *$p < 0.05$ and n.s. denotes not significant (Mann–Whitney U test).

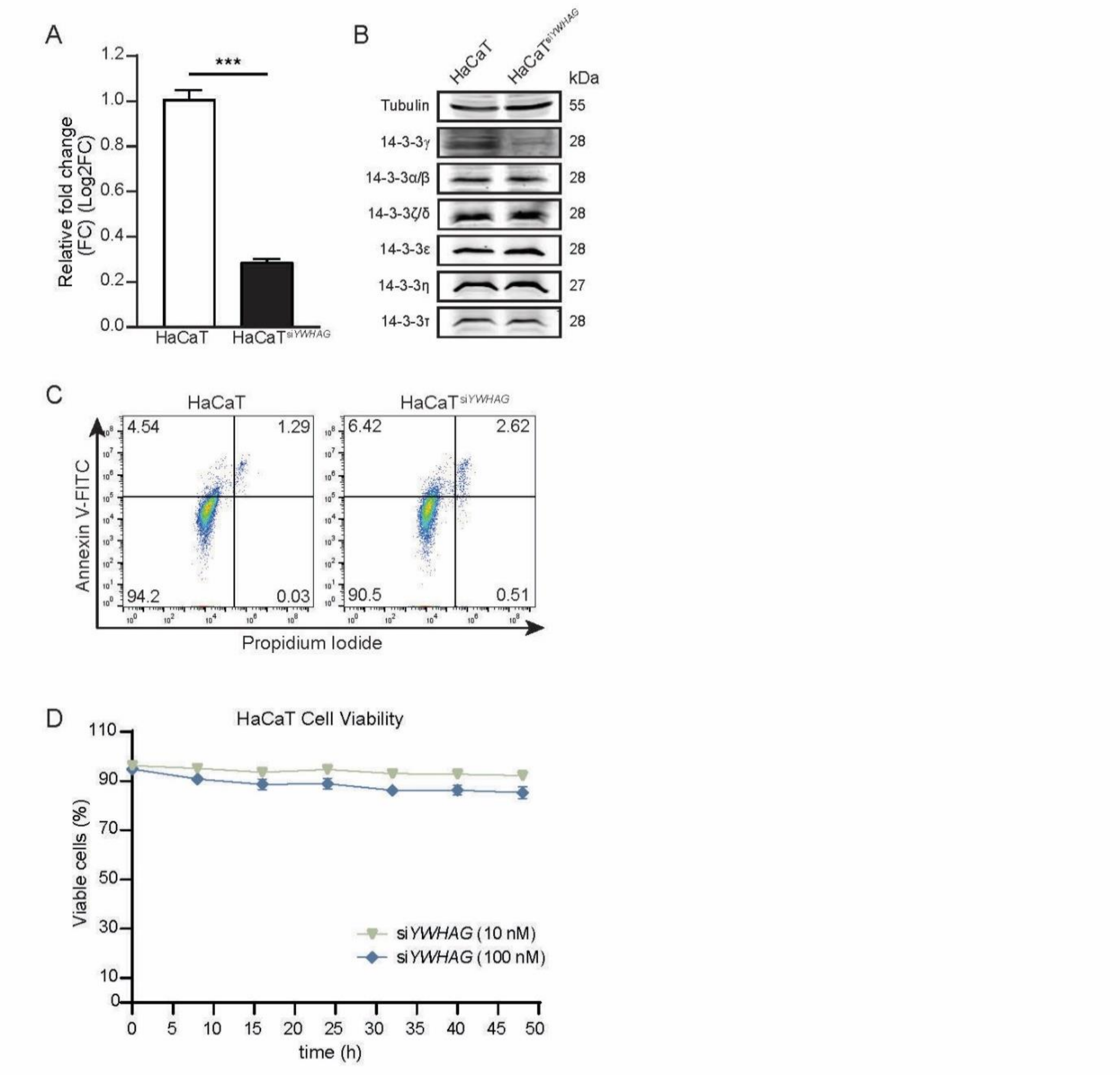

Figure S10. *YWHAG* deficiency in HaCaT cells.

(A) Relative fold change in *YWHAG* mRNA levels in HaCaT and HaCaT$^{si\mathit{YWHAG}}$ cells.

(B) Immunoblot analysis of 14-3-3γ and the other 14-3-3 isoforms in HaCaT and HaCaT$^{si\mathit{YWHAG}}$ cells.

(C) Apoptosis assay by flow cytometry using Annexin V-FITC and PI double staining in HaCaT and HaCaT$^{si\mathit{YWHAG}}$ cells.

(D) Cell viability of HaCaT and HaCaT$^{si\mathit{YWHAG}}$ cells over a period of 48 h at 8-hour intervals.

Data, wherever applicable, are represented as the mean ± SD from at least three independent experiments. ***p < 0.001, **p < 0.01, *p < 0.05 and n.s. denotes not significant (Mann–Whitney U test).

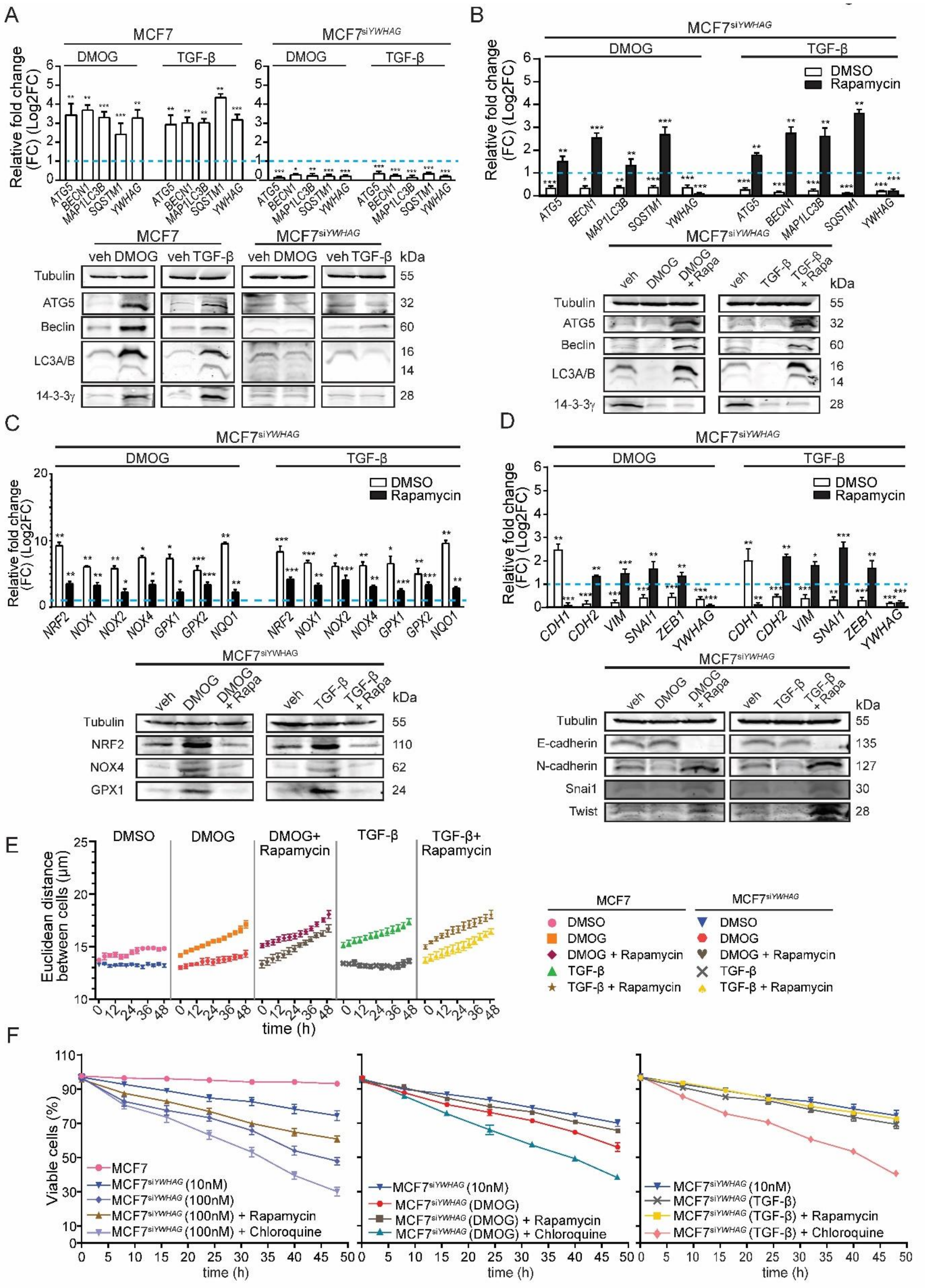
A
B
C
D
E
F
MCF7
MCF7siYWHAG
DMOG
TGF-β
DMSO
Rapamycin
Relative fold change (FC) (Log2FC)
Tubulin
ATG5
Beclin
LC3A/B
14-3-3γ
NRF2
NOX4
GPX1
E-cadherin
N-cadherin
Snai1
Twist
kDa
Euclidean distance between cells (µm)
time (h)
Viable cells (%)

Figure S11. Rapamycin-induced autophagy stimulates EMT in MCF7$^{si\textit{YWHAG}}$ cells.

(A) Relative fold change in mRNA levels (top) and immunoblot analysis (bottom) of autophagy markers (ATG5, Beclin, LC3, SQSTM1) in MKN74 cells treated with DMOG and TGF-β.

(B-D) Relative fold change in the mRNA (top) and protein (bottom) levels of autophagic (B), oxidative (C), and EMT (D) markers and *YWHAG* in untreated and rapamycin-treated MCF7$^{si\textit{YWHAG}}$ cells during DMOG- or TGF-β-induced EMT.

(E) Cell–cell Euclidean distance of MCF7 and MCF7$^{si\textit{YWHAG}}$ cells challenged with rapamycin during DMOG- or TGF-β-induced EMT.

(F) Percentage of viable MCF7 and MCF7$^{si\textit{YWHAG}}$ cells given rapamycin or chloroquine during DMOG- or TGF-β-induced EMT over 48 h at 8-h intervals.

In (A) to (D), the blue dotted lines represent the relative expression of untreated cells. Data are represented as the mean±SD from at least three independent experiments. ***$p < 0.001$, **$p < 0.01$, *$p < 0.05$ and n.s. denotes not significant (Mann–Whitney U test).

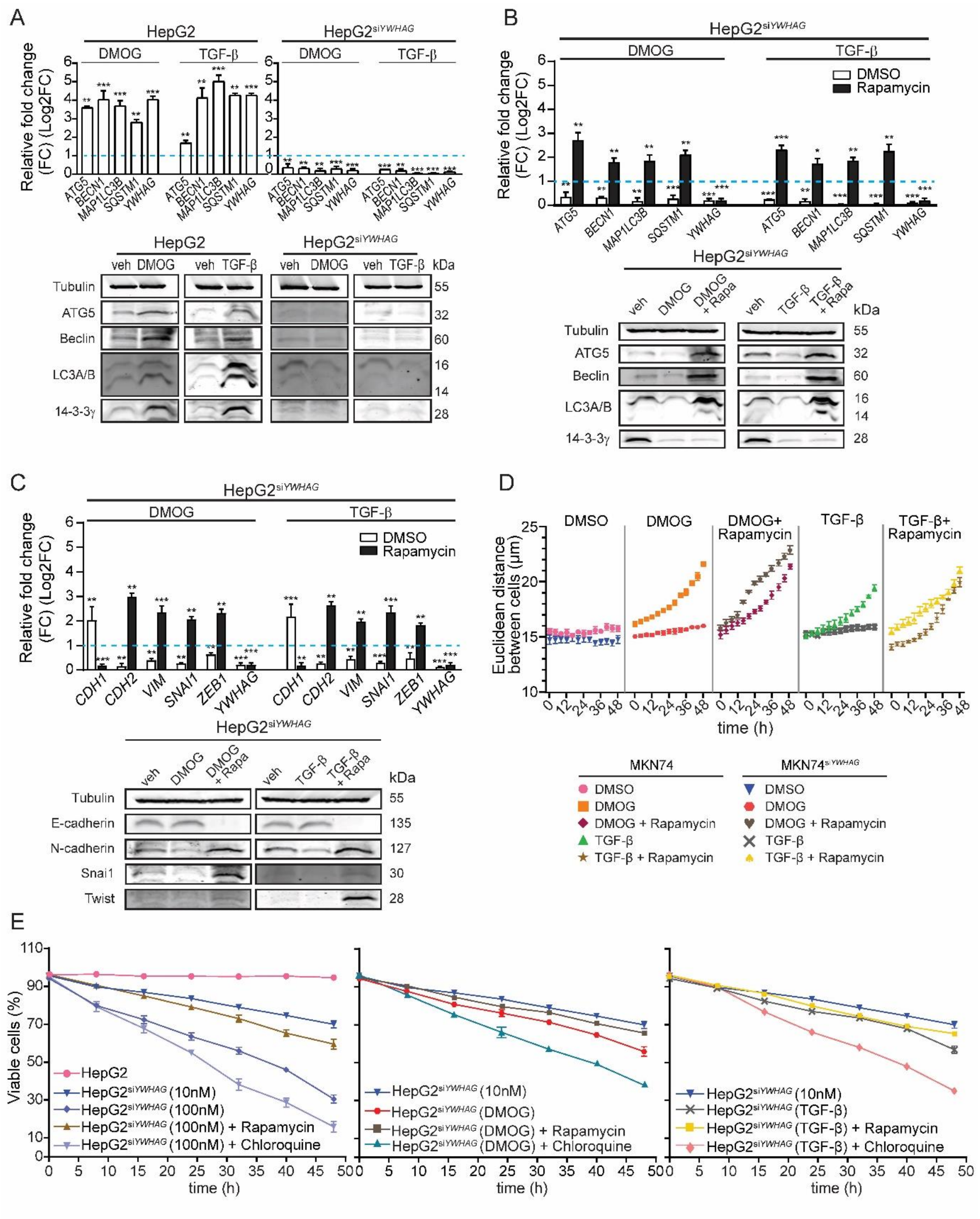

A
HepG2
HepG2siYWHAG
DMOG
TGF-β
Relative fold change (FC) (Log2FC)
ATG5
BECN1
MAP1LC3B
SQSTM1
YWHAG
veh
kDa
Tubulin
Beclin
LC3A/B
14-3-3γ
B
DMSO
Rapamycin
DMOG + Rapa
TGF-β + Rapa
C
CDH1
CDH2
VIM
SNAI1
ZEB1
E-cadherin
N-cadherin
Snai1
Twist
D
Euclidean distance between cells (µm)
time (h)
MKN74
MKN74siYWHAG
DMOG + Rapamycin
TGF-β + Rapamycin
E
Viable cells (%)
HepG2siYWHAG (10nM)
HepG2siYWHAG (100nM)
HepG2siYWHAG (100nM) + Rapamycin
HepG2siYWHAG (100nM) + Chloroquine
HepG2siYWHAG (DMOG)
HepG2siYWHAG (DMOG) + Rapamycin
HepG2siYWHAG (DMOG) + Chloroquine
HepG2siYWHAG (TGF-β)
HepG2siYWHAG (TGF-β) + Rapamycin
HepG2siYWHAG (TGF-β) + Chloroquine

Figure S12. Rapamycin-induced autophagy stimulates EMT in HepG2$^{si\mathit{YWHAG}}$ cells.

(A) Relative fold change in mRNA levels (top) and immunoblot analysis (bottom) of autophagy markers (ATG5, Beclin, LC3, SQSTM1) in HepG2 cells treated with DMOG and TGF-β.

(B-C) Relative fold change in the mRNA (top) and protein (bottom) levels of autophagic (B) and EMT (C) markers and *YWHAG* in untreated and rapamycin-treated HepG2$^{si\mathit{YWHAG}}$ cells during DMOG- or TGF-β-induced EMT.

(D) Cell–cell Euclidean distance of HepG2 and HepG2$^{si\mathit{YWHAG}}$ cells challenged with rapamycin during DMOG- or TGF-β-induced EMT.

(E) Percentage of viable HepG2 and HepG2$^{si\mathit{YWHAG}}$ cells given rapamycin or chloroquine during DMOG- or TGF-β-induced EMT over 48 h at 8-h intervals.

In (A) to (C), the blue dotted lines represent the relative expression of untreated cells. Data are represented as the mean±SD from at least three independent experiments. ***p < 0.001, **p < 0.01, *p < 0.05 and n.s. denotes not significant (Mann–Whitney U test).

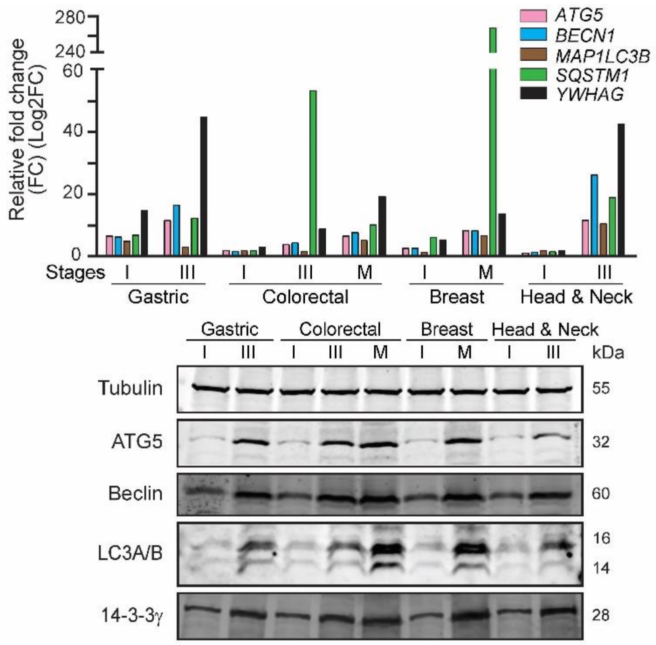

Figure S13. Expression of *YWHAG* and selected autophagic markers in human tumours.

Relative fold change in mRNA (top) and protein (bottom) of autophagy markers and *YWHAG* in breast, colorectal, gastric, and head and neck tissues at various tumour stages (stages I, II and III; metastatic M). *TBP* and *18S* were used as housekeeping genes for RT–PCR. Representative immunoblots are shown. β-tubulin was used as a loading and transfer control for immunoblot analysis from the same samples.
